# Inhibition of neutrophil extracellular trap formation ameliorates neuroinflammation and neuronal apoptosis via STING-dependent IRE1α/ASK1/JNK signaling pathway in mice with traumatic brain injury

**DOI:** 10.1186/s12974-023-02903-w

**Published:** 2023-09-30

**Authors:** Guihong Shi, Liang Liu, Yiyao Cao, Guangshuo Ma, Yanlin Zhu, Jianye Xu, Xu Zhang, Tuo Li, Liang Mi, Haoran Jia, Yanfeng Zhang, Xilei Liu, Yuan Zhou, Shenghui Li, Guili Yang, Xiao Liu, Fanglian Chen, Baolong Wang, Quanjun Deng, Shu Zhang, Jianning Zhang

**Affiliations:** 1 Department of Neurosurgery, Tianjin Medical University General Hospital, Tianjin, 300052 People’s Republic of China; 2 Key Laboratory of Post-Trauma Neuro-Repair and Regeneration in Central Nervous System, Tianjin Key Laboratory of Injuries, Variations and Regeneration of Nervous System, Tianjin Neurological Institute, Ministry of Education, Tianjin, 300052 People’s Republic of China; 3 Department of Neurosurgery, School of Medicine, Tianjin First Central Hospital, Nankai University, Tianjin, 300192 China; 4 School of Medicine, Nankai University, Tianjin, 300192 China

**Keywords:** Neutrophil extracellular traps, Cl-amidine, Neuroinflammation, Neuronal apoptosis, STING, IRE1α, Traumatic brain injury

## Abstract

**Background:**

Neuroinflammation is one of the most important pathogeneses in secondary brain injury after traumatic brain injury (TBI). Neutrophil extracellular traps (NETs) forming neutrophils were found throughout the brain tissue of TBI patients and elevated plasma NET biomarkers correlated with worse outcomes. However, the biological function and underlying mechanisms of NETs in TBI-induced neural damage are not yet fully understood. Here, we used Cl-amidine, a selective inhibitor of NETs to investigate the role of NETs in neural damage after TBI.

**Methods:**

Controlled cortical impact model was performed to establish TBI. Cl-amidine, 2′3′-cGAMP (an activator of stimulating Interferon genes (STING)), C-176 (a selective STING inhibitor), and Kira6 [a selectively phosphorylated inositol-requiring enzyme-1 alpha [IRE1α] inhibitor] were administrated to explore the mechanism by which NETs promote neuroinflammation and neuronal apoptosis after TBI. Peptidyl arginine deiminase 4 (PAD4), an essential enzyme for neutrophil extracellular trap formation, is overexpressed with adenoviruses in the cortex of mice 1 day before TBI. The short-term neurobehavior tests, magnetic resonance imaging (MRI), laser speckle contrast imaging (LSCI), Evans blue extravasation assay, Fluoro-Jade C (FJC), TUNEL, immunofluorescence, enzyme-linked immunosorbent assay (ELISA), western blotting, and quantitative-PCR were performed in this study.

**Results:**

Neutrophils form NETs presenting in the circulation and brain at 3 days after TBI. NETs inhibitor Cl-amidine treatment improved short-term neurological functions, reduced cerebral lesion volume, reduced brain edema, and restored cerebral blood flow (CBF) after TBI. In addition, Cl-amidine exerted neuroprotective effects by attenuating BBB disruption, inhibiting immune cell infiltration, and alleviating neuronal death after TBI. Moreover, Cl-amidine treatment inhibited microglia/macrophage pro-inflammatory polarization and promoted anti-inflammatory polarization at 3 days after TBI. Mechanistically, STING ligand 2′3′-cGAMP abolished the neuroprotection of Cl-amidine via IRE1α/ASK1/JNK signaling pathway after TBI. Importantly, overexpression of PAD4 promotes neuroinflammation and neuronal death via the IRE1α/ASK1/JNK signaling pathway after TBI. However, STING inhibitor C-176 or IRE1α inhibitor Kira6 effectively abolished the neurodestructive effects of PAD4 overexpression after TBI.

**Conclusion:**

Altogether, we are the first to demonstrate that NETs inhibition with Cl-amidine ameliorated neuroinflammation, neuronal apoptosis, and neurological deficits via STING-dependent IRE1α/ASK1/JNK signaling pathway after TBI. Thus, Cl-amidine treatment may provide a promising therapeutic approach for the early management of TBI.

**Graphical Abstract:**

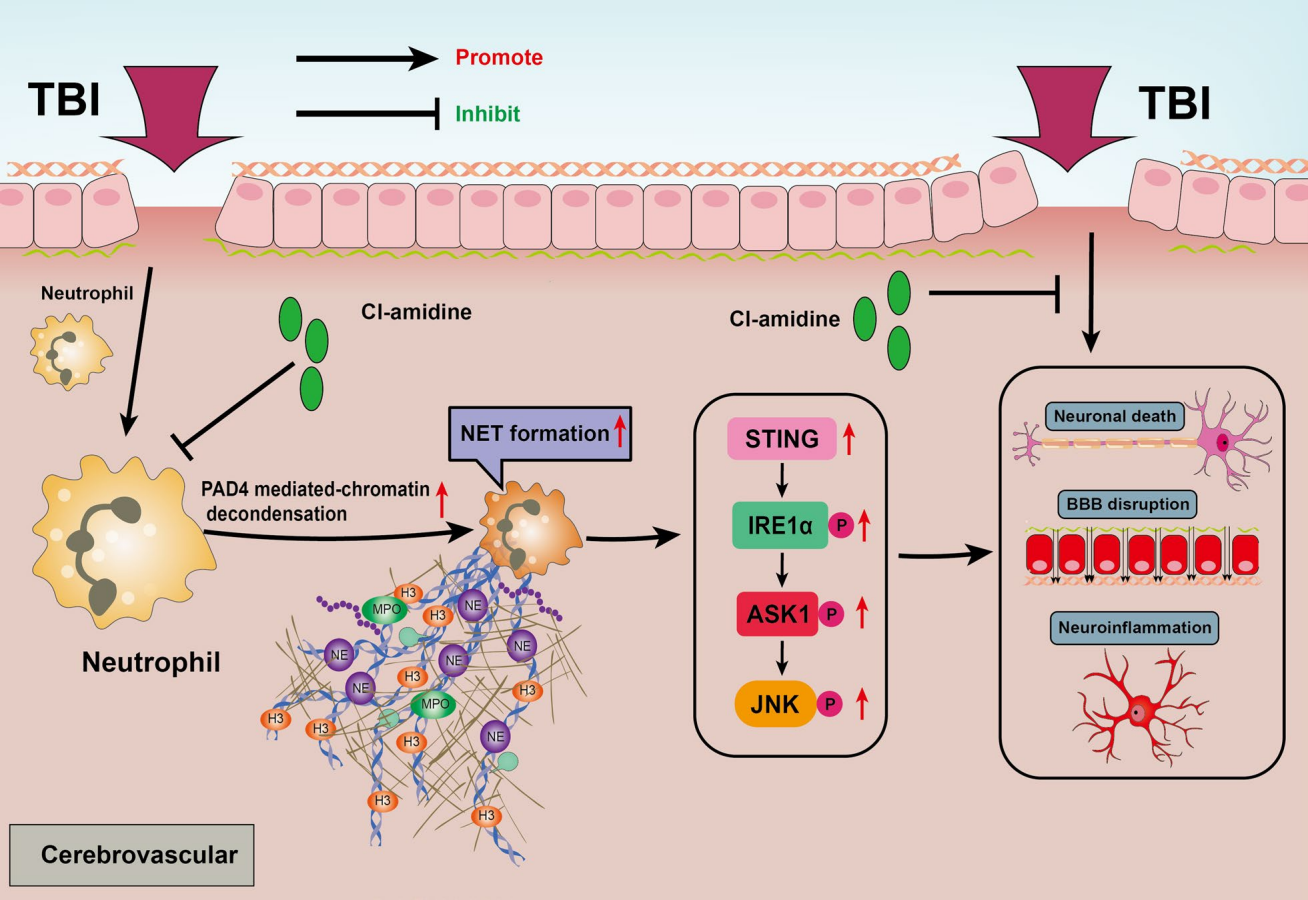

**Supplementary Information:**

The online version contains supplementary material available at 10.1186/s12974-023-02903-w.

## Introduction

Traumatic brain injury (TBI) remains one of the leading causes of death and devastating diseases worldwide across all age groups [1]. The treatment of TBI is particularly challenging because it is heterogeneous in nature and often induced various damages and complex pathogenesis pathways, which are usually classified as primary and secondary injuries. Secondary brain injury refers to further damage due to complicated and interrelated pathophysiological processes that include but are not limited to cellular calcium homeostasis, glutamate excitotoxicity, oxidative stress, neuronal apoptosis, neuroinflammatory response, and blood–brain barrier (BBB) disruption [2–4]. Recent evidence suggests that a non-resolving neuroinflammatory response and delayed neuronal injury following TBI are critical secondary injury pathogenesis that can result in chronic motor and cognitive function deficits [5]. However, therapeutic options for posttraumatic neuroinflammation and ongoing neuronal death remain limited and are often ineffective, indicating a lack of understanding of the pathophysiological mechanism of secondary brain injury post-TBI and targeted treatment approaches.

Previous studies have suggested that neutrophils as the most abundant leukocytes in circulation and the first peripheral immune cells to reach the contused brain are highly involved in regulating neuroinflammatory responses and neuronal apoptosis after brain injury [6]. Various mechanisms of neutrophil-mediated immune responses have been proposed, including neutrophil extracellular traps (NETs), an extracellular web-like structure released by neutrophils, which have been recently identified as a novel mechanism contributing to exacerbation of inflammation [7], the onset of autoimmune disorders [8] and thrombi formation [9]. Since NETs were first reported by Brinkman in 2004, the NET formation has been reported to be involved in the pathophysiological processes of various brain injuries, such as TBI [10]. NETs are composed of extracellular double-stranded DNA combined with several components, including histones, neutrophil elastase, myeloperoxidase (MPO), and cathepsin [11]. NETs have been found in the brain parenchyma [12] and cerebrovascular thrombi [13] of ischemic stroke patients and inhibition of NETs formation could improve stroke outcomes [12]. Functionally, a recent study reported increased NET in the plasma of patients with severe TBI, which correlates with elevated ICP and worse neurological function [10]. However, whether NETs forming neutrophils are involved in exacerbating neuroinflammatory responses and neuronal apoptosis after TBI remains unclear. Likewise, the underlying mechanism by which NET formation plays a detrimental role in the acute phase of TBI has yet to be determined.

DNA released by NETs is one of the main sources of circulating free DNA after TBI, and it plays a biological role by activating a series of DNA sensors in the cytosol. Cyclic guanosine 5′-monophosphate (GMP)–adenosine 5′-monophosphate (AMP) synthase (cGAS) is a crucial cytoplasmic DNA receptor that recognizes endogenous and exogenous double-stranded DNA (dsDNA) [14]. cGAS then catalyzes a reaction between guanosine triphosphate (GTP) and adenosine triphosphate (ATP) to produce a second messenger called cyclic GMP-AMP (2′3′-cGAMP), which activates the adaptor protein stimulating Interferon genes (STING) [15]. STING is a vital transmembrane dimeric protein that is located on the endoplasmic reticulum (ER) and triggers the tank-binding kinase 1 (TBK1)/ I interferon regulatory factor 3 (IRF3)/ type I interferons (IFNs) signaling pathway and proinflammatory cytokines in response to pathogenic DNA [15]. Recently studies have revealed that STING not only provokes the TBK1/IRF3/IFNs pathway in a classical way but also is involved in the regulation of ER stress and unfolded protein response (UPR) [16–18]. STING has been well reported to play an important role in regulating the neuroinflammatory process and neuronal death after TBI [19, 20], but mechanistic regulators of STING activation in TBI are currently unclear. Likewise, the underlying mechanism by which STING regulates the inflammatory process and neuronal death after TBI has yet to be explored. Interestingly, Inositol-requiring enzyme-1 alpha (IRE1α), one of the key sensor proteins in the endoplasmic reticulum associated with ER stress has been well-reported to broadly regulate neuroinflammation and neuronal death via TNF receptor-associated factor 2 (TRAF2)/apoptosis signal-regulating kinase 1(ASK1) / c-Jun N-terminal kinase (JNK) axis after TBI [21]. Consequently, STING-mediated ER stress and STING-dependent IRE1α/ASK1/JNK signaling pathway may exert neurodestructive effects in the acute phase of TBI.

In the present study, controlled cortical impact (CCI), one of the most widely applied models of TBI in animal studies, was conducted in mice to mimic clinical TBI [22]. Inspired by Vaibhav et al. Cl-amidine, an active enzyme peptidyl arginine deiminase 4 (PAD4) inhibitor was used to suppress NET formation after CCI [10]. We assumed that NET formation exacerbates neurological deficits, and promotes neuroinflammation and neuronal cell death via the STING-dependent IRE1α/ASK1/JNK signaling pathway after induction of TBI in mice.

## Materials and methods

### Animals

Male C57BL/6 mice (8–10 weeks old and 25–30 g) were purchased from the Experimental Animal Laboratories of the Academy of Military Medical Sciences (Beijing, China). Before animal experiments, the mice were acclimated in a room with controlled humidity (50–60%) and temperature (20–24℃), with a 12 h/12 h light/dark cycle and with food and water available ad libitum for at least 1 week. All animal protocols were conducted strictly in accordance with the National Institutes of Health Guide for the Care and Use of Laboratory Animals and approved by Tianjin Medical University Animal Care and Use Committee.

### Experimental design

All experimental mice were randomly assigned to the following experimental groups in the present study; all outcomes were performed by independent investigators blinded to procedures and mice groups. The experimental design was shown in Part 1 of the Additional file 1: Fig.S1.

### TBI model

TBI was conducted using a digital electromagnetically controlled cortical impact (CCI) device (eCCI-6.3 device, Custom Design & Fabrication, USA) as we previously described [23]. In brief, mice were anesthetized with 1.5–3% isoflurane in 30% oxygen and 70% nitrous oxide, placed on a heating blanket to maintain body temperature at 37 ± 0.5 °C, and subsequently mounted in a stereotaxic head frame. After exposing the skull, a circular craniotomy (3.5 mm in diameter) was performed over the right parietotemporal skull (2.5 mm posterior from the bregma and 2.5 mm lateral to the sagittal suture) using a motorized drill. The CCI model was conducted with a 3-mm flat-tip impactor and the impact parameters were set as follows: 5 m/s velocity, 200 ms dwelling time, and 2.2 mm depth. Subsequently, the mice were placed on a heating blanket to maintain their body temperature to recover from the anesthesia. The sham-operated mice were only subjected to the same craniotomy without CCI infliction.

### Drug administration

A stock solution of the PAD inhibitor, Cl-amidine (GC35706, GLPBIO, USA), was dissolved in dimethyl sulfoxide (DMSO) and diluted in saline (5% v/v). Then, 50 mg/kg Cl-amidine or vehicle (saline containing 5% DMSO) was intraperitoneally (i.p.) administered once per day for three consecutive days beginning 1 h after TBI, as reported previously [10]. 1 mg/kg 2′3′-cGAMP (tlrl-nacga23-1; InvivoGen, San Diego, USA) or vehicle (phosphate-buffered saline, PBS) was administered intravenously 10 min before TBI as previously reported [24, 25]; this was repeated 24 h and 48 h after TBI until the mice were killed. The STING antagonist C-176 (S6575, Selleck, USA) was dissolved in a stock solution containing 5% DMSO and 95% corn oil and then diluted in saline (5% v/v). As reported previously [20], 10 mg/kg C-176 or vehicle (saline containing 5% stock solution) was administered i.p. 1 h after TBI and then every day until the mice were killed. The IRE1α inhibitor Kira6 (S8658, Selleck, USA) was dissolved in a stock solution containing 5%DMSO, 30% Tween-80, and 65% corn oil and then diluted in saline (5% v/v). As previously reported [26], 10 mg/kg Kira6 or an equal volume of vehicle (saline containing 5% stock solution) was administered i.p. 1 h after TBI and once per day for three consecutive days. All inhibitors were administrated by investigators blinded to experimental groups and all inhibitors were coded by blinded investigators.

### Injection of adenoviruses

Recombinant PAD4 adenovirus (Ad-PAD4; Adeno-CMV-MCS-Padi4-3FLAG- SV40-EGFP, serotype Ad5, 4 × 10^10^ plaque-forming-unit/mL) and empty control adenovirus (Ad-con, Adeno-CMV-MCS-3-FLAG-SV40-EGFP) were produced by Genechem (Shanghai, China). The previous research has shown that after intracerebral injection of adenovirus, the peak expression of the target protein occurs between 2 and 4 days [27, 28]. Our current research is primarily focused on the acute phase after TBI (1–3 days). To better understand the role of PAD4 in the acute phase of TBI, we chose to administer the adenovirus 1 day before the CCI. As previously described [29], a total of 4 μL of Adeno-PAD4 or Adeno-EGFP was stereotactically injected into two different locations in the right cortex at a dose of 2 μL each (1.5 mm caudal and 1.5 mm rostral from the lesion epicenter) at a depth of 1.5 mm using a 5 μL Hamilton syringe (Hamilton Company, USA) at a 0.5 μL/min flow rate through a hole. After injection, the needle was maintained in the position for 15 min before retraction, and then the scalp was sutured. The injection of adenovirus was conducted 1 day before mice were subjected to CCI and was analyzed 4 days after injection. All adenovirus were injected by investigators blinded to experimental groups and all adenovirus were coded by blinded investigators.

### Neurological score assessment

Neurological function was assessed using the modified neurological severity score (mNSS), as described previously [30]. Following TBI, the mNSS of mice was measured on days 1 and 3. The mNSS consists of motor (muscular state and abnormal action), sensory (visual and tactile), reflex, and balance tests. One point is scored for each abnormal behavior or untested reflex, with an overall score of 0 being normal, 1–6 mild, 7–12 moderate, and 13–18 severe. See details in Additional file 1: Table S1 in Part 2 of the Additional file 1: Table S1. The assessment was performed by two individuals blinded to the group identity of each mouse.

### Corner test

Sensorimotor and postural asymmetries were evaluated by the corner turn test [31]. Before the test is started, ensure mice are acclimated to the testing environment for at least 30 min and verify that the corner apparatus is clean and free from odors. Briefly, mice were placed between two plastic boards positioned at a 30° angle facing the corner and exited by turning either to the left or the right. Record the direction the mouse turns when it reaches the corner. Repeat the test a minimum of 10 times for each mouse, with a 30-s interval between trials. Sham mice turned either side at equal frequencies whereas mice subjected to TBI would preferentially turn to the ipsilateral direction. The data was presented as the percentage of right turns based on the formula: number of right turns/total number of turns) × 100%.

### Cylinder test

The Cylinder test is a valuable behavioral assessment tool for evaluating forelimb motor function in mouse models of traumatic brain injury (TBI) [32]. Before the test is started, allow mice to acclimate to the testing environment for at least 30 min and maintain consistent lighting conditions during testing. The Cylinder test was carried out in a transparent cylinder (9 cm in diameter and 38 cm in height). A camera was placed over the cylinder for 10 min recording after a mouse was placed in. Record the frequency of forelimb (left/right/both) contacts made by the mouse with the cylinder walls during the test period from the video. As previously described, the data was calculated based on the formula: (right−left)/(left + right + both) × 100% [33].

### Rotarod test

As described previously [34], the limb motor coordination and balance of mice were assessed using an accelerating Rota-rod apparatus (RWD Life Science, Shenzhen, China). Before induction of CCI, Mice were exposed to a period of acclimation and training (first acclimation session: 0 rpm for 30 s followed by three training sessions) every day of three trials each for three consecutive days: mice were trained at a slow rotational speed (4 rpm/min) for 1 min followed by an accelerating rotational speed (from 4 to 40 rpm in 5 min) with periods of 30 min between trials. Then, at 1 day and 3 days post-TBI, each mouse was placed on the accelerating automated Rota-rod, which accelerated from 4 to 40 rpm/min within 5 min. The latency to fall for each mouse was then recorded. Each mouse was tested three times with the same speed each day with an interval of 30 min between trials, and the average latency to fall was used for analysis. The test was performed by two investigators blinded to the animal groups assignments.

### Magnetic resonance imaging

Magnetic resonance imaging (MRI) scanning was used to measure brain edema on a 9.4T 30 cm bore BioSpec MRI spectrometer (Bruker Biospec 94/30USR, Billerica, MA) at 1 and 3 days after CCI. The setup parameters were as follows: repetition time, 4000 ms; echo time, 35 ms; field of view, 40 × 40 mm^2^; image matrix, 256 × 256; slice thickness, 0.5 mm. The hyperintensity signal in T2-weighted (T2W) images at and around the site of cortical impact as edema caused by CCI [35]. All hyperintensities were quantified by measuring the T2-hyperintense area using Weasis software [36]. All analyses were done by investigators blinded to experimental groups.

### Laser speckle contrast imaging

To assess the cerebral blood flow (CBF) changes in the cortex at pre-surgery and at 1 h, 12 h, 1 day, and 3 days after CCI, we used a laser speckle contrast imaging (LSCI) system (PeriCam PSI System, Perimed AB, Sweden) as we previously described [23]. Briefly, the mice were anesthetized and placed prone in a stereotaxic head frame. Then, a midline incision was made over the skull to expose the calvaria. The exposure area was kept clean and dry using a tampon during image collection. The CBF was continuously measured for 60 s at the following settings: observation height, 10 cm; laser irradiation area, 2 × 2 cm; image matrix, 1388 × 1038. We select the whole cerebral cortex (100 mm^2^) as the region of interest (ROI). The CBF changes were evaluated using the vendor-supplied PIMsoft software (version 1.4; Perimed) [23]. The measurement and analysis of laser speckle were done by investigators blinded to experimental groups.

### Quantification of plasma DNA

Plasma was collected from the whole blood of mice by centrifugation at 500 ×*g* for 20 min. DNA in plasma was quantified according to the manufacturer’s instructions of the Quant-iT PicoGreen dsDNA Assay kit (P11496, Invitrogen).

### Quantification of NETs

An *in-house* ELISA was used to quantify MPO-DNA complexes. First, Plasma was collected from the whole blood of mice by centrifugation at 500 ×*g* for 20 min at 4 ℃; mouse cerebral cortex samples were homogenized in the lysis buffer (1:10 dilution) and centrifugated at 1500 rpm for 15 min at 4 ℃ to collect the supernatant. Briefly, after overnight coating with anti-MPO antibody (1:500, 0400–0002, Bio-Rad) at 4 ℃, 96-well plates were then blocked with 5% BSA for 2 h at room temperature. After washing three times (300 μL), 50 μL of plasma or 50 μL of tenfold dilution of the mouse brain homogenates was added into the wells with 80 μL of incubation buffer containing a peroxidase-labeled anti-DNA mAb (Cell Death ELISA, 11774425001, Roche, Basel, Switzerland). The plates were then incubated at room temperature for 2 h. After five washes, the plate was developed with 100 μL ABST substrate. The absorbance (OD) at 450 nm was measured after 30 min incubation in the dark. All samples for completion and analysis of ELISA were performed by two researchers blinded to experimental groups.

### MPO activity assay

As described previously [37], the ipsilateral brain cortex was homogenized in 50 mM potassium phosphate buffer, centrifuged, and then resuspended in 0.5% cetyltrimethylammonium bromide (Sigma-Aldrich) in potassium phosphate buffer. After centrifugation, 40 μL of supernatant was incubated with 100 μL tetramethylbenzidine solution (Sigma-Aldrich) in a 96-well plate and the reaction was stopped with 100 μL 2 N HCl. The absorbance (OD) at 450 nm was measured after 10 min incubation in the dark. MPO activity was in equivalent units by comparison with a reference curve generated using purified MPO (Sigma-Aldrich) according to the manufacturer’s instructions. All samples for completion and analysis of ELISA were performed by two researchers blinded to experimental groups.

### Evans blue extravasation assay

The BBB permeability was assessed by the extravasation of Evans blue dye (EB) as previously described [38]. The 2% Evans blue (4 mL/kg, Sigma Aldrich) in sterile saline was injected intravenously and circulated in the mice for 2 h before the mice were sacrificed. The mice were transcardially perfused with PBS, and their brains were dissected and weighed. The samples were then homogenized in n, n-dimethylformamide (Sigma Aldrich), and incubated at 60 °C for 72 h. After centrifugation, the absorption of the supernatant was determined by spectrophotometry (Molecular Devices, Sunnyvale, CA) at optical density (OD) 620 nm. The sample collection and ELISA analysis were performed by two researchers in a blinded manner.

### Quantitative real-time polymerase chain reaction (qPCR)

Total RNA was extracted from the homogenized contused cortex 3 days after TBI using Trizol regent (Invitrogen, Thermo fisher scientific), and cDNA was synthesized from 2 μg RNA using PrimeScript RT Reagent Kit (Takara Bio, Tokyo, Japan) according to the manufacturer’s instructions. The PCR assays were processed on a Real-Time PCR Detection System (Bio-Rad, Hercules, CA, USA) using SYBR Green PCR Master Mix (Applied Biosystems, Waltham, MA, USA). GAPDH was used as an internal control. The relative quantitation value for each gene was performed using the comparative cycle threshold method [39]. The primers used in this study are shown in Part 2 of the Additional file 1: Table S2. The RNA extraction and PCR analysis were performed by two investigators in a blinded manner.

#### Fluoro-Jade C staining

The degenerating neurons were evaluated by FJC staining using a modified FJC Ready-to-Dilute Staining Kit (Millipore, Billerica, MA, USA) 3 days after TBI as previously described [40]. According to the manufacturer's instructions, the slides were washed by PBS with FJC working solution for 20 min and then visualized using an inverted fluorescence microscope (Olympus, Japan). FJC-positive neurons were manually counted in the both cortex and hippocampus per brain using ImageJ software (Version 1.46r, Wayne Raband, USA). The data were averaged and expressed as FJC-positive cells/mm^2^. The section per brain were obtained by two researchers blinded to animal groups assignments. In addition, the FJC-positive cells were counted in all sections per brain in a blinded manner with a fluorescence microscope.

#### TUNEL staining

For observation of apoptotic neurons, double staining of the neuronal marker NeuN (red) and TUNEL (green) was conducted using the In Situ Cell Death Detection kit (Roche, South San Francisco, CA, USA) according to the manufacturer’s instructions[22]. The nuclei were counterstained with DAPI (Abcam) and imaged by an inverted fluorescence microscope (Olympus, Japan). In the contused area, TUNEL-positive neurons were manually counted in the both cortex and hippocampus using ImageJ software (Version 1.46r, Wayne Raband, USA). The data were expressed as TUNEL-positive neurons/mm2. The sections’ completion and TUNEL-positive neuron count analysis were performed in a blinded manner.

#### Western blot analysis

Western blotting was performed as previously described [22]. Equal amounts of protein were suspended in a loading buffer (denatured at 100 ℃ for 15 min) and were then separated by sodium dodecyl sulfate–polyacrylamide gel electrophoresis (SDS-PAGE), and finally transferred to a 0.45 μm pore size polyvinylidene difluoride membranes (Millipore, Temecula, CA, USA). The membrane was blocked and then incubated overnight at 4 °C with the following primary antibodies: rabbit anti-Histone H3 (anti-H3; 1:1000, 9715, Cell Signaling Technology, USA), rabbit anti-STING (1:1000, 13647, Cell Signaling Technology, USA), rabbit anti-IL-1β (1:500, 12703, Cell Signaling Technology, USA), rabbit anti-Arginase-1(1:500, 93668, Cell Signaling Technology, USA), rabbit anti-Bcl-2 (1:1000, 3498, Cell Signaling Technology, USA), rabbit anti-Bax (1:1000, 14796, Cell Signaling Technology, USA), rabbit anti-pJNK (1:1000, 9255, Cell Signaling Technology, USA), rabbit anti-JNK (1:1000, 9252, Cell Signaling Technology, USA), rabbit anti-Caspase-3 (1:1000, 9664, Cell Signaling Technology, USA), rabbit anti-IgG (1:1000, 14708, Cell Signaling Technology, USA), rabbit anti-H3Cit (1:1000, ab5103, Abcam, UK), rabbit anti-PAD4 (1:1000, ab96758, Abcam, UK), rabbit anti-VE-cadherin (1:1000, ab205336, Abcam, UK), rabbit anti-ZO-1 (1:5000, ab276131, Abcam, UK), rabbit anti-Occludin (1:1000, ab216327, Abcam, UK), goat anti-Iba-1 (1:2000, ab5076, Abcam, UK), rabbit anti-pIRE1α (1:1000, ab48187, Abcam, UK), rabbit anti-IRE1α (1:1000, ab37073, Abcam, UK), rabbit anti-pASK1(1:1000, ab278547, Abcam, UK), rabbit anti-ASK1(1:1000, ab45178, Abcam, UK), rat anti-Ly6G (1:1000, 551459, BD Pharmingen), CD16 (1:500, MA5-36143, Invitrogen), CD206 (1:100, sc-58986, Santa Cruz Biotechnology). The membranes were incubated with mouse anti-β-actin (1:5000, 3700, Cell Signaling Technology, USA) as a loading control. Thereafter, the membranes were then washed with TBST and incubated with species-appropriate horseradish peroxidase (HRP)-labeled secondary antibodies (1:5000, Cell Signaling Technology, USA) for 1 h at room temperature. Finally, the immunoblot bands were visualized under an imaging system (Bio-Rad, Hercules, CA, USA) and were qualified using ImageJ software (Version 1.46r, Wayne Raband, USA). All protein samples for completion and analysis of immunoblots were performed by two researchers blinded to experimental groups.

#### Immunofluorescence staining

After being anesthetized deeply after CCI, mice were transcardially perfused with ice-cold PBS followed by whole brains removed quickly and fixed in 4% paraformaldehyde (PFA) at 4 ℃ for 24 h. The brains were removed and sliced into 8 μm-thick coronal sections using a cryostat (Leica, Model CM1950, Germany). Sections were washed with PBS and permeabilized with 0.1% Triton X-100 (Sigma Aldrich) for 30 min and incubated with 3% BSA for 1 h at room temperature. Thereafter, sections were incubated overnight at 4 °C with the following primary antibodies: including rat anti-F4/80 (1:200, ab6640, Abcam, UK), goat anti-Iba1 (1:500, ab5076, Abcam, UK), rabbit anti-MPO (1:500, ab208670, Abcam, UK), rabbit anti-H3Cit (1:500, ab5103, Abcam, UK), rabbit anti-ZO-1 (1:5000, ab276131, Abcam, UK), rabbit anti-NeuN (1:1000, ab117487, Abcam, UK), rabbit anti-pIRE1α (1:500, ab48187, Abcam, UK), goat anti-GFAP (1:200, ab53554, Abcam, UK), goat anti-CD31 (1:200, AF806, R&D Systems), goat anti-MPO (1:500, AF3667, all from R&D Systems), rabbit anti-Arginase-1(1:500, 93668, Cell Signaling Technology, USA), rabbit anti-IL-1β (1:500, 12703, Cell Signaling Technology, USA), rat anti-mouse CD16/32(1:500, 553142, BD Biosciences), rat anti-Ly6G (1:200, 551459, BD Pharmingen). The sections were then incubated with the species-appropriate Alexa Fluor-conjugated IgG (1:500, Invitrogen, USA) for 1 h at room temperature. Nuclear staining was performed with 4ʹ,6-diamidino-2-phenylidole (DAPI, Abcam). For the purpose of measuring neuronal loss across different brain regions, we conducted an analysis of the percentage of neuronal reactive area in four selected anatomical fields on three coronal sections. These regions encompassed the cortex (CTX), hippocampus CA1, hippocampus CA3, and hippocampus DG (Fig. 6B). As previous studies described [41, 42], brain tissue, including the hippocampus, can be highly heterogeneous with variations in cell density. Counting cells in such tissue can be challenging, whereas measuring the reactive area takes into account these density differences. Therefore, we used the percentage of reactive area in order to determine the tissue area that was immunoreactive. Images were changed to binary code and the positive staining was used to set the threshold, the optical density was automatically determined and the percentage of stained area was calculated over the total area of the frame which was for all cases of 1.21 mm^2^ (1.1 mm × 1.1 mm). For the CTX region, the region of interest (ROI) in the NeuN reactive area is the entire field micrograph image, while the region of interest (ROI) in the three hippocampal regions was based on the positive areas within their respective anatomical boundaries. For other immunostaining analyses, three coronal sections were randomly chosen, displaying the ipsilateral cortex and hippocampus. The results were presented as the mean number of cells per mm^2^ for single staining or expressed as the number of cells for the main marker double-labeled with the neurons, microglia, or neutrophils per mm^2^ for double-staining. All images were captured using an inverted fluorescence microscope (Olympus, Japan) with a 10 × eyepiece and a 20 × objective or 40 × objective, and we set the same exposure time when quantitative analysis was involved in each experiment. Images were analyzed using ImageJ software (Version 1.46r, Wayne Raband, USA). The relative immunofluorescence intensity of ZO-1 was calculated by the percentage of immunofluorescence intensity of ZO-1 relative to the immunofluorescence intensity of CD-31 with ImageJ software (Version 1.46r, Wayne Raband, USA). All sections acquisition for completion of immunofluorescence and analysis of quantitative fluorescence outcomes were performed by two researchers blinded to experimental groups.

### Statistical analysis

The normality of all data in this study was evaluated with the Shapiro–Wilk test. All data in this study are expressed as means ± SD with dots. All data analysis was performed using Graph-Pad Prism software (Graph Pad Software, Version 8.1.2 San Diego, CA, USA) and was conducted by an experimenter blinded to the experimental conditions. Multiple comparisons were analyzed by one-way analysis of variance (ANOVA) followed by Tukey’s multiple comparison post hoc test. All statistical tests were two-sided; a p < 0.05 was defined as statistically significant. To assess the significance of group differences and interaction effects of CBF changes after TBI, we conducted a two-way analysis of variance (ANOVA). The results of the overall ANOVA are as follows: Interaction Effect (Time X Group): F (F (8, 84) = 10.23, p < 0.001. To further investigate the main effects of our independent variables, we conducted separate one-way ANOVAs for Time and Groups as follows: Main effect (Time): F (2.978, 62.54) = 148.2, p < 0.001; Main effect (Group): F (2, 21) = 95.8, p < 0.001. The p-values associated with these tests were all < 0.05. In cases where the overall ANOVA and main effects were significant, we conducted Tukey’s multiple comparison post hoc test to identify specific group differences.

## Results

### Neutrophils form NETs presenting in the brain after TBI

**Fig. 1:**
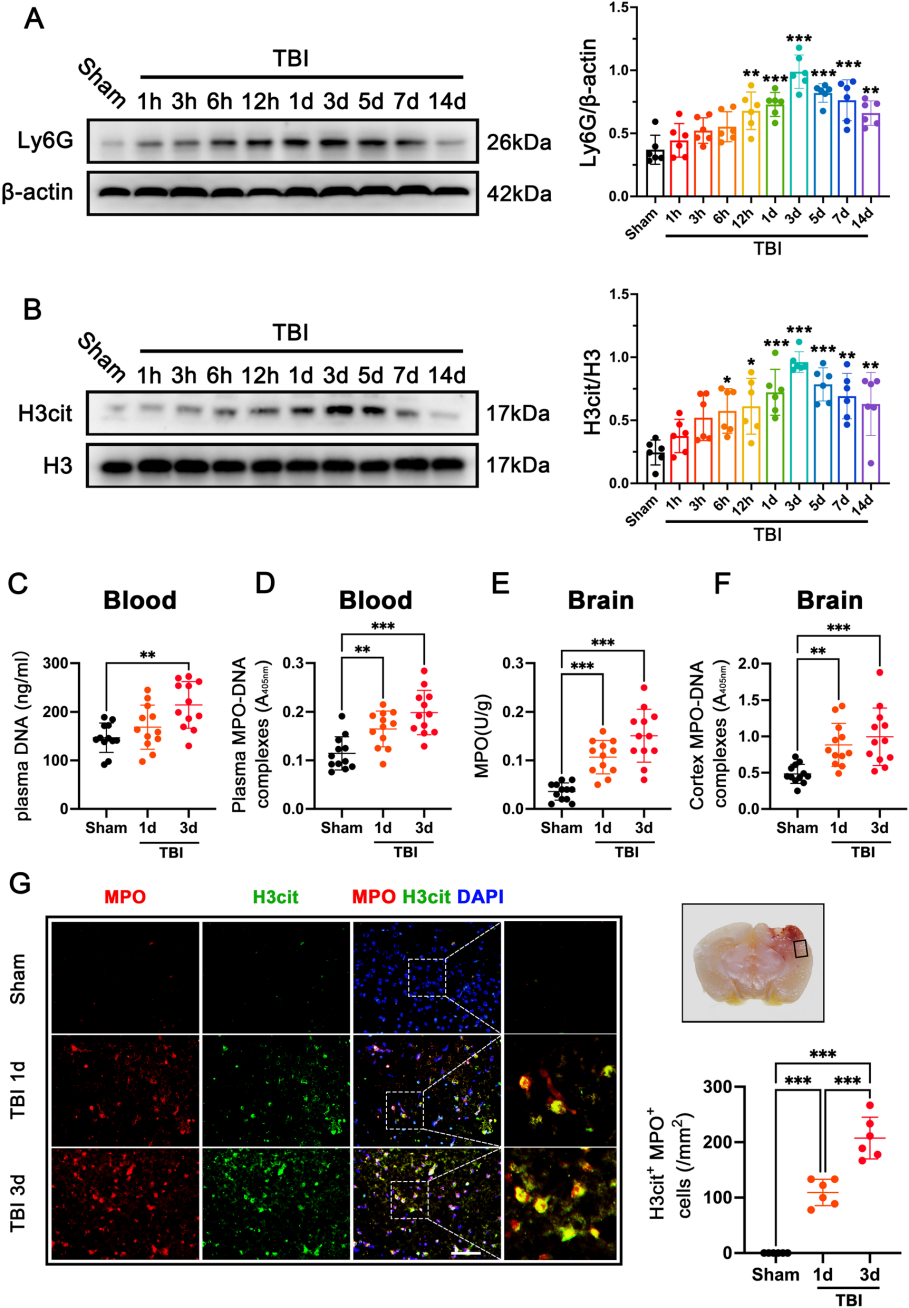
Neutrophils form NETs presenting in the circulation and brain after TBI. **A** Representative western blot bands of the time course of Ly6G and densitometric quantification of Ly6G after TBI. **p < 0.01, ***p < 0.001, n = 6 per group. **B** Representative western blot bands of the time course of H3cit and densitometric quantification of H3cit after TBI. **p < 0.01, ***p < 0.001, n = 6 per group. **C** Quantitative analyses of plasma DNA at 1 day and 3 days after TBI. **p < 0.01, n = 12 per group. **D** Quantitative analyses of plasma MPO-DNA complexes at 1 day and 3 days after TBI. **p < 0.01, ***p < 0.001, n = 12 per group. **E** Quantification of MPO activity in the brain of mice at 1 day and 3 days after TBI. ***p < 0.001, n = 12 per group. **F** Quantitative analyses of cortex MPO-DNA complexes at 1 day and 3 days after TBI. **p < 0.01, ***p < 0.001, n = 12 per group. **G** Representative images of the colocalization of H3cit (green) with neutrophils (MPO, red) and quantitative analysis of H3cit-positive neutrophils at the lesion site at 1 days and 3 days after TBI. Nuclei were stained with DAPI (blue). Scale bar = 50 μm, ***p < 0.001, n = 6 per group

We subjected the mice to TBI and analyzed the brain cortexes at 1 h, 3 h, 6 h, 12 h, 1 day, 3 days, 5 days, 7 days, and 14 days. Using an anti-lymphocyte antigen 6 complex locus G (Ly6G) antibody, we found that the neutrophil level in the ipsilateral cerebral cortex increased in a time-dependent manner compared with sham mice, reaching the peak level at 3 days after TBI (p < 0.001; Fig. 1A**)**. Consistently, the expression of NETs marker H3cit in the ipsilateral cortex increased in a time-dependent manner compared with that in sham-operated mice, reaching the peak level at 3 days (p < 0.001; Fig. 1B**)** and persisting for at least 14 days after TBI **(**Fig. 1B**).** Then, we found that the plasma DNA levels were significantly elevated in the plasma of TBI mice when compared with sham-operated mice at 3 days post-TBI (p < 0.01; Fig. 1C). ELISA analysis also showed a marked increase in the total content of the MPO-DNA complex in the plasma from TBI mice compared with sham-operated mice, especially at 3 days (1.44-fold increase) post-TBI (p < 0.001; Fig. 1D**)**. Consistently, we then found a marked increase in total content of the neutrophil enzyme MPO in the cortical areas at 1 day and 3 days after TBI (p < 0.001, Fig. 1E**)**. ELISA analysis also showed a marked increase in the total content of the MPO-DNA complex in the cortical homogenates from TBI mice compared with sham-operated mice at 1 day (p < 0.01, Fig. 1F) and 3 days (p < 0.001, Fig. 1F**)** post-TBI. To validate these observations, double immunofluorescence staining further showed that H3cit-positive neutrophils in the contused cortex were also significantly increased at 1 day and 3 days after TBI (p < 0.001, Fig. 1G). Peptidyl arginine deiminase 4 (PAD4) is a key histone-modifying enzyme that plays a key role in NET formation [43]. Consistent with the expression of Ly6G and H3cit in the contused cortex of mice with TBI, we found that the expression of PAD4 in the ipsilateral cortex increased in a time-dependent manner compared with that in sham-operated mice, reaching the peak level at 3 days (p < 0.001; Additional file 1: Fig. S2A). Further, double immunofluorescence staining showed that PAD4-positive neutrophils in the contused cortex were also significantly increased at 1 day and 3 days after TBI (p < 0.001, Additional file 1: Fig. S2B).

### PAD4 inhibitor Cl-amidine ameliorated short-term neurological deficits and attenuated cerebral lesion volume, brain edema, and CBF decrease after TBI

To establish the role of NETs in secondary brain injury after TBI, we administrated the TBI mice with PAD4 inhibitor Cl-amidine for three consecutive days beginning 1 h after TBI. We found that Cl-amidine treatment substantially decreased the expression of H3cit in the contused cortex after TBI (p < 0.01, Additional file 1: Fig. S2C). Consistently, we found that Cl-amidine treatment substantially reduced neutrophil NETs, as seen by the decrease in H3cit-positive neutrophils in the cortex (p < 0.01, Additional file 1: Fig. S2D) and plasma DNA levels (p < 0.01, Additional file 1: Fig. S2E) in the circulation at 3 days after TBI.

**Fig. 2:**
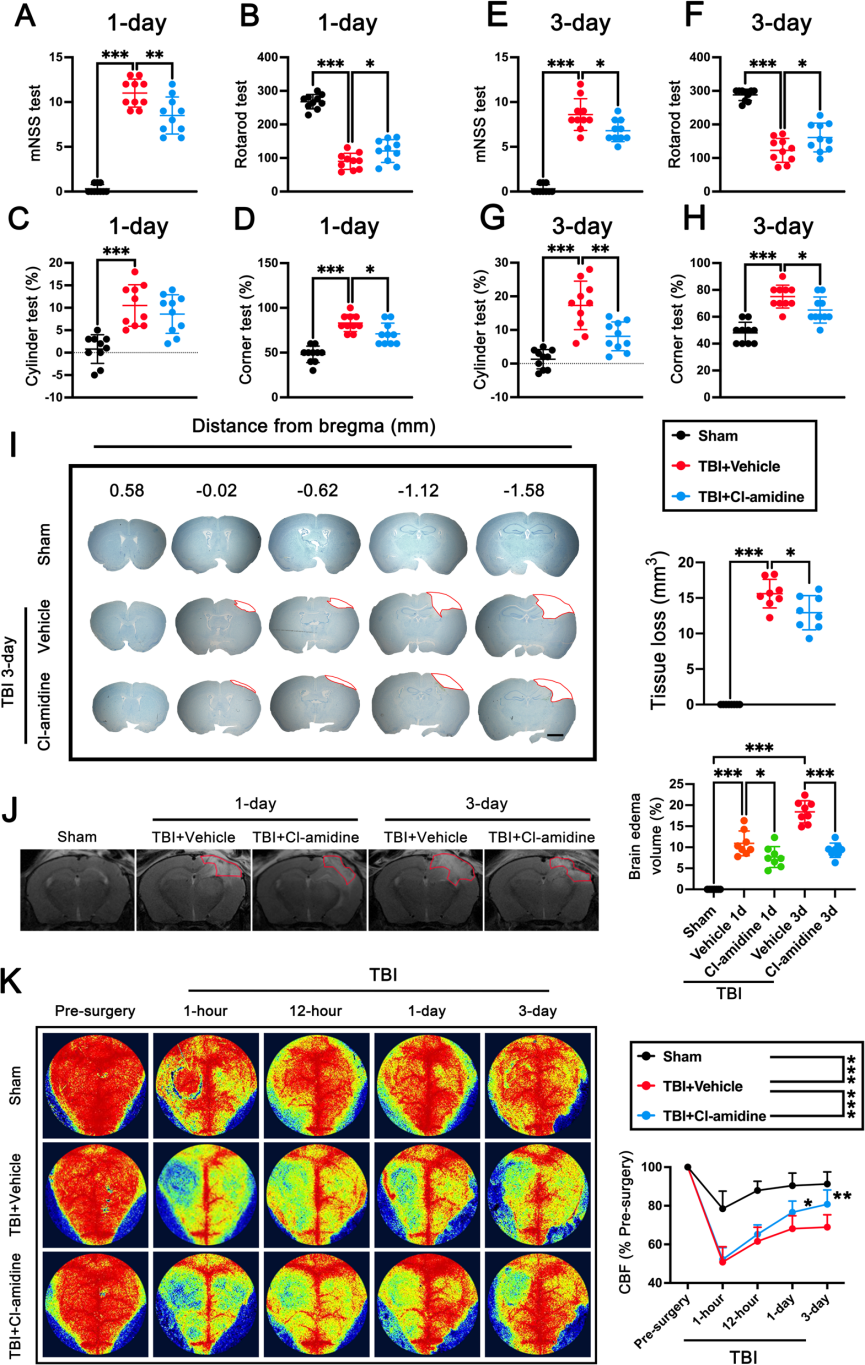
The effects of Cl-amidine treatment on short-term neurological functions, cerebral lesion volume, brain edema, and CBF after TBI. **A**–**D** mNSS test (**A**), Rotarod test (**B**), Cylinder test (**C**), and Corner test (**D**) at 1 day post-TBI. *p < 0.05, **p < 0.01, ***p < 0.001, n = 10 per group **E**–**H** mNSS test (**E**)**,** Rotarod test (**F**), Cylinder test (**G**), and Corner test (**H**) at 3 days post-TBI. *p < 0.05, **p < 0.01, ***p < 0.001, n = 10 per group. **I** Representative images of serial coronal sections labeled by Nissl and quantitative analysis of tissue loss at 3 days after TBI. Scale bar = 1 mm, *p < 0.05, ***p < 0.001, n = 8 per group. **J** Representative images of MRI scanning at 1 day and 3 days after TBI and quantitative analysis of brain edema volume at 1 day and 3 days after TBI. *p < 0.05 ***p < 0.001, n = 8 per group. **K** Representative images of CBF by LSCI in different groups at different time points after TBI and quantitative analysis of continuous CBF changes before and after CCI. *p < 0.05, **p < 0.01, ***p < 0.001, n = 8 per group

To determine whether Cl-amidine treatment influenced the recovery of short-term neurological functions in mice after TBI, the mNSS test, Rotarod test, Cylinder test, and Corner test were performed. The results showed that mice in the TBI + Vehicle group exhibited marked defects in neurological function compared with those of mice in the sham group on days 1 and 3 post-TBI, as assessed by mNSS test (p < 0.001, Fig. 2A, E), Rotarod test (p < 0.001, Fig. 2B, F), Cylinder test (p < 0.001, Fig. 2C, G), and Corner test (p < 0.001, Fig. 2D, H), indicating the TBI model induce by CCI was successfully established. However, Cl-amidine treatment significantly ameliorated neurological deficits assessed by the mNSS test at 1 day (p < 0.01, Fig. 2A) and 3 days (p < 0.05, Fig. 2E), Rotarod test at 1 day (p < 0.05, Fig. 2B) and 3 days (p < 0.05, Fig. 2F), Cylinder test at 3 days (p < 0.01, Fig. 2G), and Corner test at 1 day (p < 0.05, Fig. 2D) and 3 days (p < 0.05, Fig. 2H) after TBI. Then, Nissl staining on the serial coronary sections was conducted to assess neuronal tissue loss. Results showed that the Cl-amidine treatment post-TBI significantly reduced the tissue loss at 3 days after TBI when compared with TBI + Vehicle mice (p < 0.05, Fig. 2I).

To further verify the neuroprotective effects of Cl-amidine, MRI and LSCI were performed at 1 day and 3 days post-TBI. MRI revealed that TBI caused significant brain edema at 1 day and 3 days after TBI (p < 0.001, Fig. 2J). Cl-amidine post-treatment significantly decreased the edema volume when compared with TBI + Vehicle group at 1 day (p < 0.05, Fig. 2J) and 3 days (p < 0.001, Fig. 2J). After TBI, the CBF of the whole brain was significantly decreased compared with the baseline within 3 days (p < 0.001, Fig. 2K**)**. Cl-amidine treatment significantly rescue the decreased CBF of the whole brain within 3 days after TBI (p < 0.001, Fig. 2K) when compared with TBI + Vehicle group, especially at 1 day (p < 0.05, Fig. 2K) and 3 days post-TBI (p < 0.01, Fig. 2K).

### Cl-amidine treatment attenuated BBB disruption after TBI

**Fig. 3:**
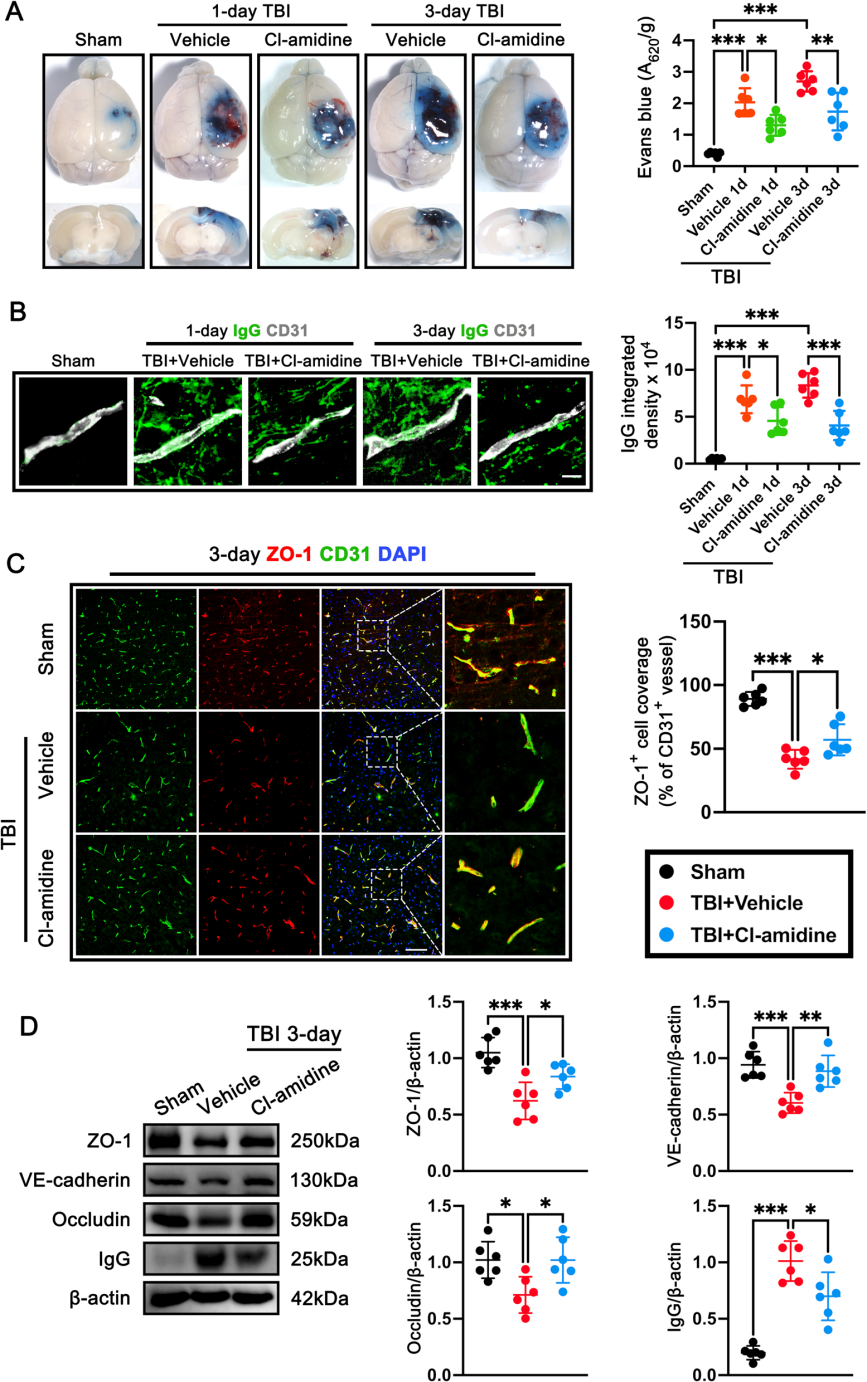
The effects of Cl-amidine treatment on BBB disruption after TBI. **A** Representative horizontal image of brains after EB injection and quantitative analysis of EB leakage intensity. *p < 0.05, **p < 0.01, ***p < 0.001, n = 6 per group. **B** Representative images and quantitation of IgG extravascular deposits (green). Capillaries were stained with CD31 (white). Nuclei were stained with DAPI (blue). Scale bar = 10 μm, *p < 0.05, ***p < 0.001, n = 6 per group. **C** Representative images of double immunofluorescence staining of ZO-1(red) and endothelial cells (CD31, green) and quantification of fluorescence intensity analysis of ZO-1 (relative to CD31) at 3 days after TBI. Nuclei were stained with DAPI (blue). Scale bar = 100 μm. *p < 0.05, ***p < 0.001, n = 6 per group. **D** Representative western blot bands and densitometric quantification of ZO-1, VE-cadherin, Occludin, and IgG at 3 days after TBI. *p < 0.05, **p < 0.01, ***p < 0.001, n = 6 per group

An increase in BBB permeability greatly aggravates secondary brain injury after TBI [44]. To investigate the role of Cl-amidine on BBB permeability, we first analyzed Evans blue (EB) dye extravasation at 0 day (Sham), 1 day, and 3 days after TBI. Results showed that EB dye leaked into brain parenchyma was significantly increased **(**Fig. 3A**)**, and EB extravasation (p < 0.001, Fig. 3A) was significantly increased in TBI mice at 1 day and 3 days when compared with the Sham group. Administration of Cl-amidine significantly decreased the EB extravasation at 1 days **(**p < 0.05, Fig. 3A) and 3 days post-TBI (p < 0.01, Fig. 3A) when compared with the TBI + Vehicle group. Immunostaining for IgG and the endothelial cell marker CD31 further confirmed a marked reduction in perivascular IgG deposits at 1 day (p < 0.05, Fig. 3B) and 3 days (p < 0.001, Fig. 3B) in Cl-amidine-treated mice. We then further determined the effects of Cl-amidine on BBB permeability at 3 days after TBI by double immunofluorescence and western blot analyses. We detected endothelial junction protein ZO-1^+^ vessels in the contused cortex were significantly reduced at 3 days after TBI (p < 0.001, Fig. 3C), whereas administration of Cl-amidine post-TBI in mice significantly increased ZO-1^+^ vessels compared with the TBI + Vehicle group (p < 0.05, Fig. 3C). These observations were further supported by the western blot, which showed that expressions of tight junction proteins ZO-1 (p < 0.001, Fig. 3D), VE-cadherin (p < 0.001, Fig. 3D), and Occludin (p < 0.05, Fig. 3D) were significantly reduced, whereas expression of IgG (p < 0.001, Fig. 3D) were significantly increased at 3 days after TBI. However, these observations were significantly reversed in mice receiving Cl-amidine (p < 0.05, Fig. 3D).

### Cl-amidine treatment inhibited microglia activation, neutrophil and macrophage infiltration, and the expression of IL‑1β at 3 days after TBI

**Fig. 4:**
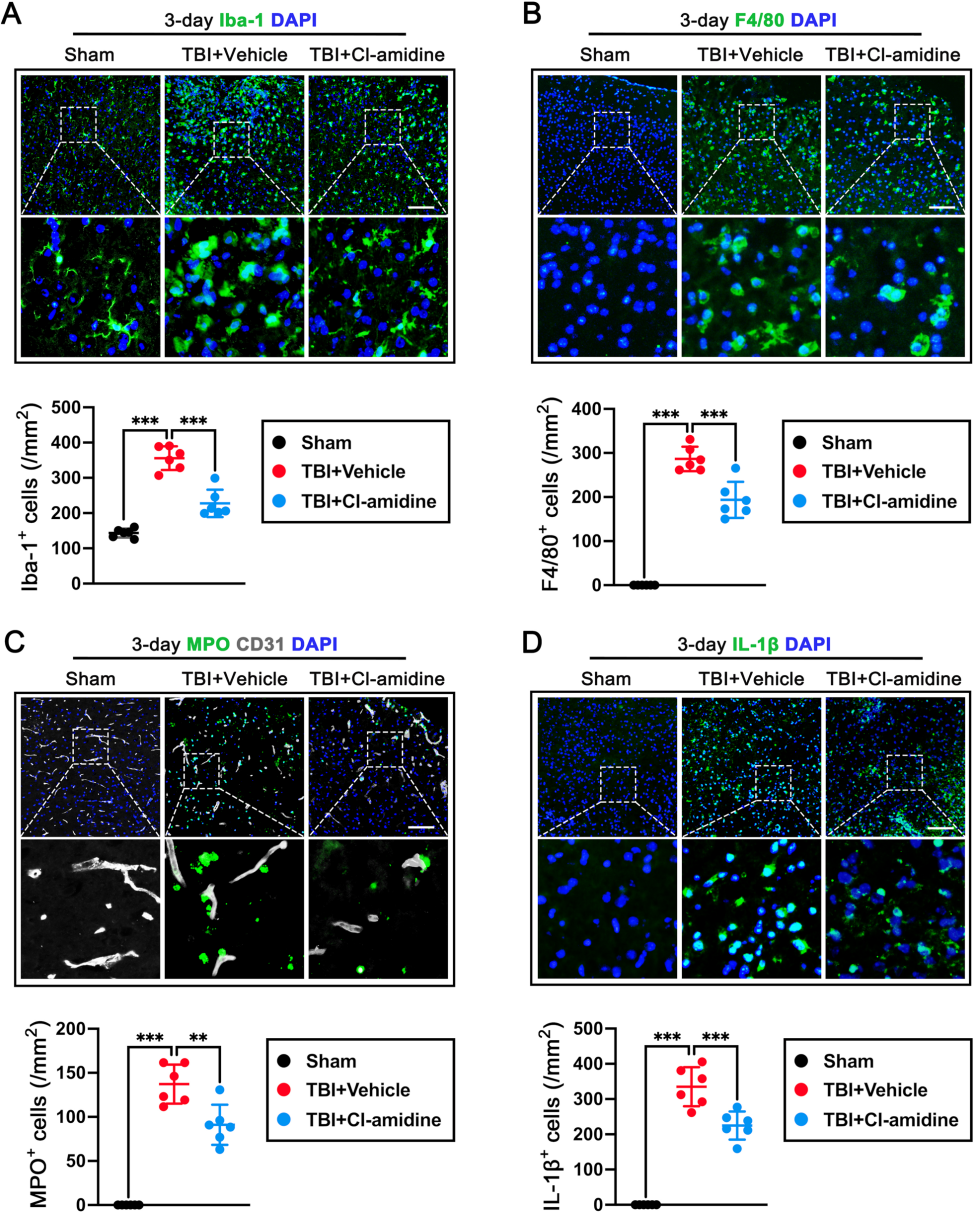
The effects of Cl-amidine treatment on microglia/macrophage activation, neutrophil, and macrophage infiltration, and the expression of IL‑1β at 3 days after TBI. **A** Representative image of immunofluorescence staining of Iba-1 (green) and quantitative analysis of Iba-1-positive microglia at 3 days after TBI. Nuclei were stained with DAPI (blue). ***p < 0.001, n = 6 per group. Scale bar = 100 μm.** B** Representative image of immunofluorescence staining of F4/80 (green) and quantitative analysis of F4/80-positive macrophages at 3 days after TBI. Nuclei were stained with DAPI (blue). ***p < 0.001, n = 6 per group. Scale bar = 100 μm. **C** Representative image of double immunofluorescence staining of MPO (green) and CD31 (grey), and quantitative analysis of MPO-positive neutrophils at 3 days after TBI. Nuclei were stained with DAPI (blue). **p < 0.01, ***p < 0.001, n = 6 per group. Scale bar = 100 μm. **D** Representative image of immunofluorescence staining of IL-1β (green) and quantitative analysis of IL-1β-positive cells at 3 days after TBI. Nuclei were stained with DAPI (blue). ***p < 0.001, n = 6 per group. Scale bar = 100 μm

To determine the effect of Cl-amidine on microglia/macrophage activation and immune cell infiltration following TBI, quantitative analyses were measured by immunofluorescence. Immunofluorescence staining showed that the number of Iba-1-positive microglia (p < 0.001, Fig. 4A), F4/80-positive macrophages (p < 0.001, Fig. 4B), MPO-positive neutrophils (p < 0.001, Fig. 4C), and IL‑1β-positive cells (p < 0.001, Fig. 4D) were significantly increased in TBI + Vehicle group when compared with the Sham group. However, Cl-amidine post-treatment significantly reduced the number of Iba-1-positive microglia (p < 0.001, Fig. 4A), F4/80-positive macrophages (p < 0.001, Fig. 4B), MPO-positive neutrophils (p < 0.01, Fig. 4C), and IL‑1β-positive cells (p < 0.001, Fig. 4D) in the contused cortex when compared with TBI + Vehicle group.

### Cl-amidine treatment promoted the phenotypic switch of microglial/macrophage from a pro-inflammatory to an anti-inflammatory phenotype at 3 days after TBI

**Fig. 5:**
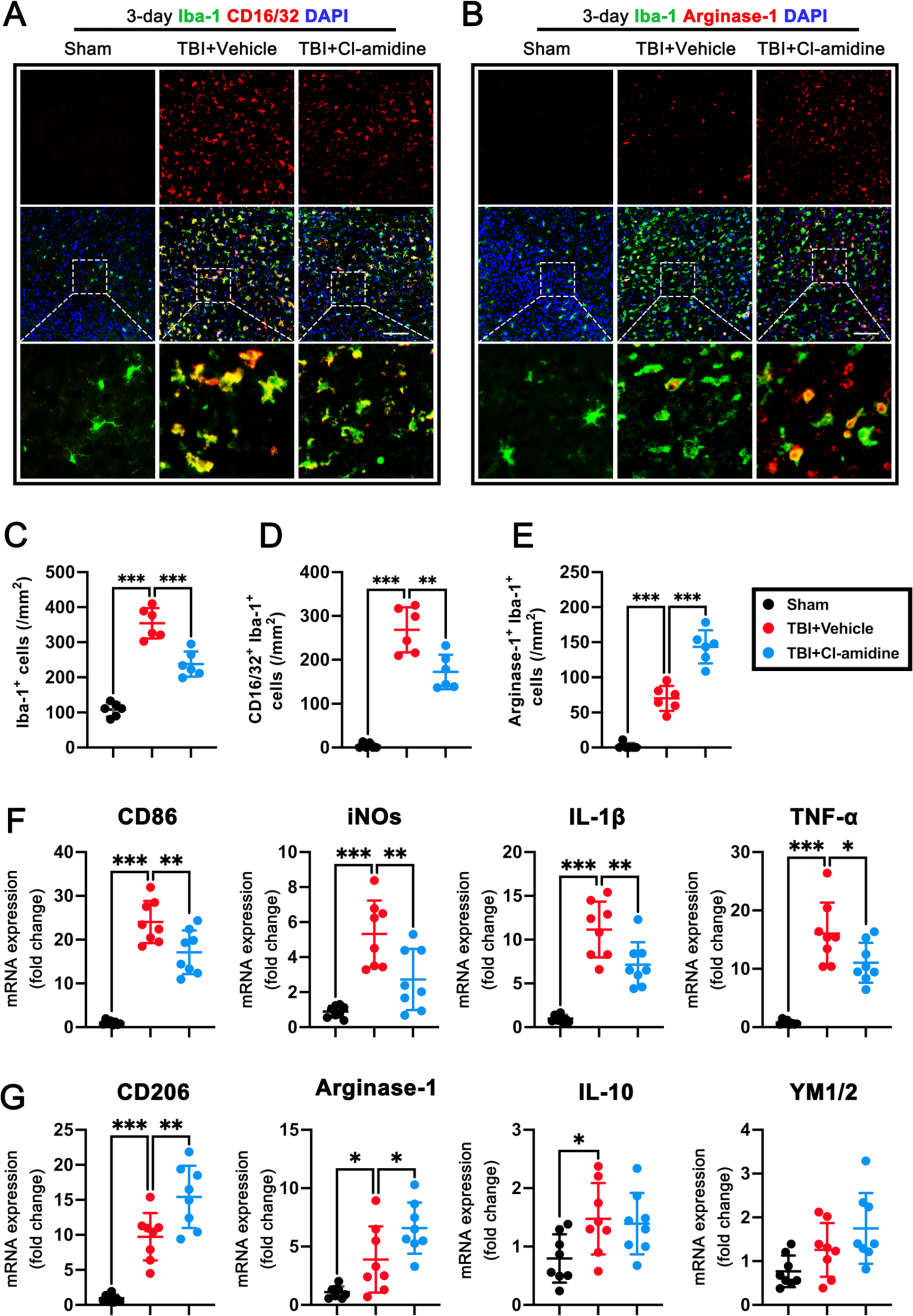
Cl-amidine promotes the phenotype of microglia/macrophage from pro-inflammatory to anti-inflammatory at 3 days after TBI. **A** Representative double immunofluorescence staining for Iba1 (green) and CD16/32 (red) in the contused cortex at 3 days after TBI. Nuclei were stained with DAPI (blue). Scale bar = 100 μm. **B** Representative double immunofluorescence staining for Iba1 (green) and Arginase-1 (red) in the contused cortex at 3 days after TBI. Nuclei were stained with DAPI (blue). Scale bar = 100 μm. **C**–**E** Quantitative analyses of Iba1^+^ microglia/ macrophage (**C**), Iba1^+^/CD16/32^+^ M1 microglia/macrophage (**D**), and Iba1^+^/Arginase-1^+^ M2 microglia/macrophage (**E**) in the contused cortex at 3 days after TBI. **p < 0.01, ***p < 0.001, n = 6 per group. **F** M1-associated mRNA levels were evaluated including CD86, iNOs, IL-1β, and TNF-α. **G** M2-associated mRNA levels were evaluated including CD206, Arginase-1, IL-10, and YM1/2. *p < 0.05, **p < 0.01, ***p < 0.001, n = 6 per group

It is well established that in response to acute brain injury, microglia/macrophages can be categorized into two polarization phenotypes: the classically activated M1-like phenotype and the alternatively activated M2-like phenotype [45]. The different phenotypes of microglia/macrophages influence the outcome of TBI. Double immunofluorescence staining and qPCR were used to validate whether Cl-amidine affects microglia/macrophage phenotypic switch. First, immunofluorescence staining showed that administration of Cl-amidine significantly reduced the numbers of Iba1-positive microglia/macrophage (p < 0.001, Fig. 5A–C) in the contused cortex at 3 days post-TBI when compared with TBI + Vehicle group, which indicated that Cl-amidine attenuated microglial activation at 3 days post-TBI. Comparing with the TBI + Vehicle group, we found that Cl-amidine post-treatment significantly reduced the numbers of CD16^+^ Iba1^+^ M1 microglia/macrophages (p < 0.01, Fig. 5A, D) and markedly increased the numbers of Arginase-1^+^ Iba1^+^ M2 microglia/macrophages (p < 0.001, Fig. 5B, E) in the contused cortex at 3 days post-TBI. To further determine the phenotype characteristics of microglia/macrophages after TBI, qPCR was used to evaluate the mRNA expression levels of M1 phenotype markers (CD86, iNOs, IL-1β, and TNF-α) and M2 phenotype markers (CD206, Arginase-1, IL-10, and YM1/2). Results showed that compared with the TBI + Vehicle group, Cl-amidine treatment significantly downregulated the mRNA expression levels of M1 phenotype markers [CD86 (p < 0.01, Fig. 5F), iNOs (p < 0.01, Fig. 5F), IL-1β (p < 0.01, Fig. 5F), and TNF-α (p < 0.05, Fig. 5F)] and upregulated the mRNA expression levels of M2 phenotype markers [CD206 (p < 0.01, Fig. 5G) and Arginase-1(p < 0.05, Fig. 5G)] in the contused cortex at 3 days after TBI. Taken together, these findings indicated that Cl-amidine post-treatment promoted the phenotypic switch of microglia/macrophages from pro-inflammatory to anti-inflammatory phenotype after TBI.

### Cl-amidine treatment alleviated neuronal death at 3 days after TBI.

**Fig. 6:**
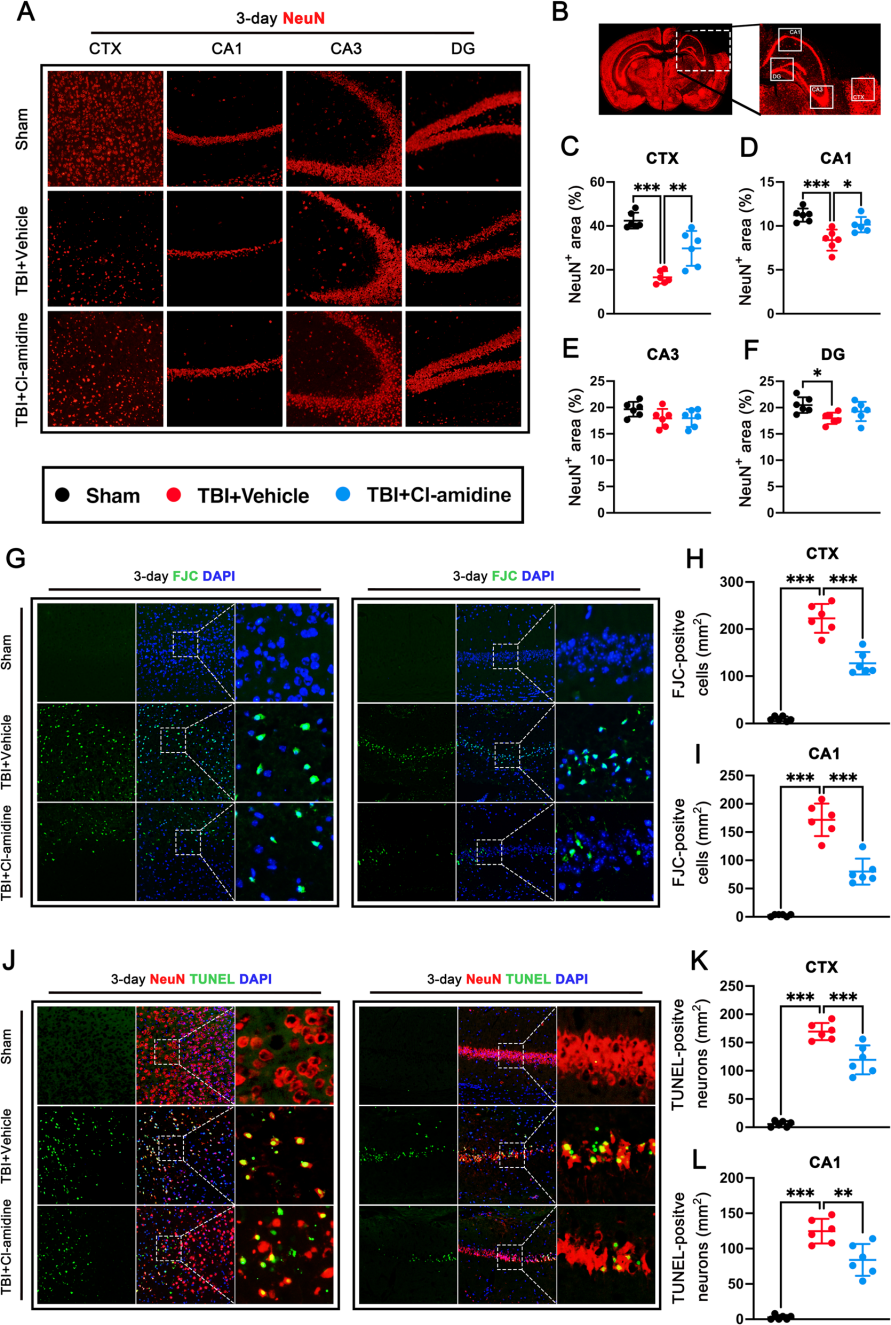
Effects of Cl-amidine treatment on neuronal death at 3 days after TBI. **A** Representative image of NeuN immunostaining in the ipsilateral CTX, CA1, CA3, and DG regions at 3 days after TBI. Nuclei were stained with DAPI (blue). Scale bar = 200 μm. **B** Ipsilateral brain areas (rectangles) in the CTX, CA1, CA3, and DG where images in A were captured. Scale bar = 1 mm. **C**–**F** Quantitative analysis of NeuN‑immunopositive neurons in the ipsilateral CTX (**C**), CA1(**D**), CA3 (**E**), and DG (**F**) regions at 3 days after TBI. **G** Representative image of the FJC (green) staining in the CTX and CA1 at 3 days after TBI. Nuclei were stained with DAPI (blue). Scale bar = 100 μm. **H**, **I** Quantitative analysis of FJC -positive cells in the CTX (**H**) and CA1 (**I**) at 3 days after TBI. ***p < 0.001, n = 6 per group. **J** Representative images of TUNEL (green) co-localization with neurons (NeuN, red) in the CTX and CA1 at 3 days after TBI. Nuclei were stained with DAPI (blue). Scale bar = 100 μm. **K**, **L** Quantitative analysis of TUNEL-positive neurons in the CTX (**K**) and CA1 (**L**) at 3 days after TBI. **p < 0.01, ***p < 0.001, n = 6 per group

To investigate the effects of Cl-amidine post-treatment in neuronal death after TBI, we examined cortical and hippocampal neuronal loss in mice 3 days after TBI by using NeuN immunofluorescence staining. NeuN staining images were taken from the ipsilateral cerebral cortex (CTX), hippocampal CA1, hippocampal CA3, and hippocampal DG regions as shown in Fig. 6A, B. Quantitative results showed that TBI induced significant neuronal loss in the ipsilateral CTX (p < 0.001, Fig. 6C), hippocampal CA1 (p < 0.001, Fig. 6D), and hippocampal DG regions (p < 0.05, Fig. 6F), but not in the hippocampal CA3 (p = 0.191, Fig. 6E) regions when compared with sham controls. However, Cl-amidine treatment significantly ameliorated neuronal loss in ipsilateral CTX (p < 0.01, Fig. 6C), hippocampal CA1 (p < 0.05, Fig. 6D), but not in the hippocampal CA3 (p = 0.999, Fig. 6E) and hippocampal DG (p = 0.316, Fig. 6F) regions compared with TBI + Vehicle group.

In parallel, degenerating and apoptotic neurons in the ipsilateral CTX and hippocampal CA1 at 3 days after TBI was assessed by FJC and TUNEL staining. FJC staining showed that FJC-positive neurons were significantly increased in both the ipsilateral CTX (p < 0.001, Fig. 6G, H).and hippocampal CA1(p < 0.001, Fig. 6G, I) of mice 3 days post-TBI. However, Cl-amidine administration significantly decreased the numbers of FJC-positive neurons in both the ipsilateral CTX (p < 0.001, Fig. 6G, H) and hippocampal CA1(p < 0.001, Fig. 6G, I) of mice 3 days post-TBI when compared with TBI + Vehicle group. Similarly, TUNEL staining showed that compared with the TBI + Vehicle group, Cl-amidine treatment significantly decreased the numbers of TUNEL-positive neurons in both the ipsilateral CTX (p < 0.001, Fig. 6J, K) and hippocampal CA1(p < 0.01, Fig. 6J, L) at 3 days after TBI.

### STING ligand 2′3′-cGAMP abolished neuroprotection of Cl-amidine via IRE1α/ASK1/JNK signaling pathway at 3 days after TBI

Western blot analysis and immunofluorescence staining were conducted to characterize the expression profile of STING after TBI. Western blot analysis was used to assess the STING expression at 0 h (Sham), 6 h, 12 h, 1 day, 3 days, 5 days, 7 days, and 14 days after CCI in the contused cortex. Analysis of the western blot bands showed that the expression of STING significantly increased in a time-dependent manner and peaked at 3 days after TBI when compared with the Sham group (p < 0.001, Additional file 1: Fig. S3A). Double immunofluorescence staining was performed to identify the cellular distribution of STING at 3 days in the ipsilateral peri-lesion cortex after TBI. The results (Additional file 1: Fig. S3B, C) showed that in the peri-lesion cortex at 3 days after TBI, STING was mainly expressed in the neurons (NeuN^+^) and microglia (Iba-1^+^), whereas STING was a little co-localized with astrocytes (GFAP^+^) and no co-localized with neutrophils (Ly6G^+^) and endothelial cells (CD31^+^). Then, we found that Cl-amidine treatment significantly decreased the expression of STING in the cortex of mice at 1 day (p < 0.001, Additional file 1: Fig. S3D) and 3 days (p < 0.001, Additional file 1: Fig. S3D) after TBI, indicating STING may participate in regulating NETs- mediated secondary brain injury.

**Fig. 7:**
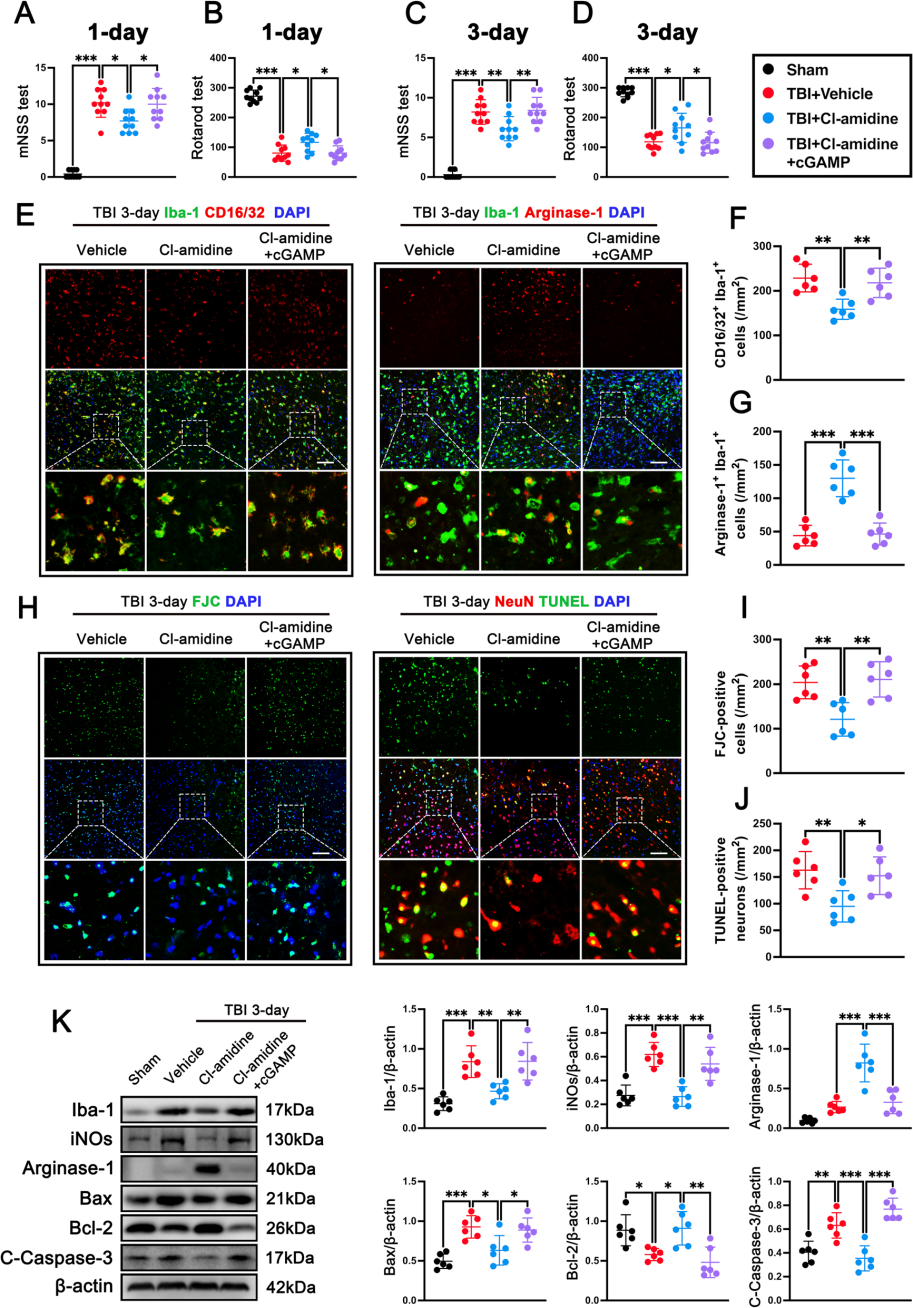
STING agonist 2′3′-cGAMP abolished the anti-inflammatory and anti-apoptotic effects of Cl-amidine after TBI. **A**, **B** mNSS test (**A**) and Rotarod test (**B**) at 1 day post-TBI. *p < 0.05, ***p < 0.001, n = 10 per group **C**, **D** mNSS test (**C**) and Rotarod test (**D**) at 3 days post-TBI. *p < 0.05, **p < 0.01, ***p < 0.001, n = 10 per group. **E** Representative double immunofluorescence staining for Iba1 (green) and CD16/32 (red) and representative double immunofluorescence staining for Iba1 (green) and Arginase-1 (red) in the contused cortex at 3 days after TBI. Nuclei were stained with DAPI (blue). Scale bar = 100 μm. **F**, **G** Quantitative analyses of Iba1^+^/CD16/32^+^ M1 microglia/macrophage (**F**), and Iba1^+^/Arginase-1^+^ M2 microglia/macrophage (**G**) in the contused cortex at 3 days after TBI. **p < 0.01, ***p < 0.001, n = 6 per group. **H** Representative image of the FJC (green) staining and representative images of TUNEL (green) co-localization with neurons (NeuN, red) in the contused cortex at 3 days after TBI. Nuclei were stained with DAPI (blue). Scale bar = 100 μm. **I**, **J** Quantitative analysis of FJC-positive cells (**I**) and quantitative analysis of TUNEL-positive neurons (**J**) in the contused cortex at 3 days after TBI. *p < 0.05, **p < 0.01, n = 6 per group.** K** Representative western blot bands and densitometric quantification of Iba-1, iNOs, Arginase-1, Bax, Bcl-2, and C-Caspase-3 after TBI. *p < 0.05, **p < 0.01, ***p < 0.001, n = 6 per group

To understand the mechanism of neuroprotection of Cl-amidine, we first analyzed whether STING activation abolished the neuroprotection of Cl-amidine, and we activated STING with a second messenger called 2′3′-cGAMP pre-injury. The primary hypothesis of this experiment is that STING, as a downstream protein of NETs, can reduce STING activation through NETs inhibition, and pre-treatment with CGAMP can advance STING’s sensitivity. If NETs mediate downstream pathway changes through STING, then the pre-treatment with CGAMP is expected to reverse the inhibitory effects induced by NETs. Interestingly, pre-administration of STING ligand 2′3′-cGAMP significantly abolished neurological benefits of Cl-amidine assessed by mNSS test at 1 day (p < 0.05, Fig. 7A) and 3 days (p < 0.01, Fig. 7C), and Rotarod test at 1 day (p < 0.05, Fig. 7B) and 3 days (p < 0.05, Fig. 7D) after TBI. Comparing with the TBI + Cl-amidine group, we found that 2′3′-cGAMP pre-administration significantly increased the numbers of CD16^+^ Iba1^+^ M1 microglia/macrophages (p < 0.01, Fig. 7E, F) and markedly reduced the numbers of Arginse-1^+^ Iba1^+^ M2 microglia/macrophages (p < 0.001, Fig. 7E, G) in the contused cortex at 3 days post-TBI. Meanwhile, we also found that TBI + Cl-amidine + 2′3′-cGAMP group significantly increased the FJC-positive cells (p < 0.01, Fig. 7H, I) and TUNEL-positive neurons (p < 0.05, Fig. 7H, J) in contused cortex when compared with TBI + Cl-amidine group at 3 days after TBI. Consistently, 2′3′-cGAMP pre-administration significantly upregulated the expression of Iba-1 (p < 0.01, Fig. 7K), iNOs (p < 0.01, Fig. 7K), Bax (p < 0.05, Fig. 7K), and Caspase-3 (p < 0.001, Fig. 7K), while downregulated the proteins levels of Arginase-1 (p < 0.001, Fig. 7K) and Bcl-2 (p < 0.01, Fig. 7K) in contused cortex when compared with TBI + Cl-amidine group at 3 days after TBI.

**Fig. 8:**
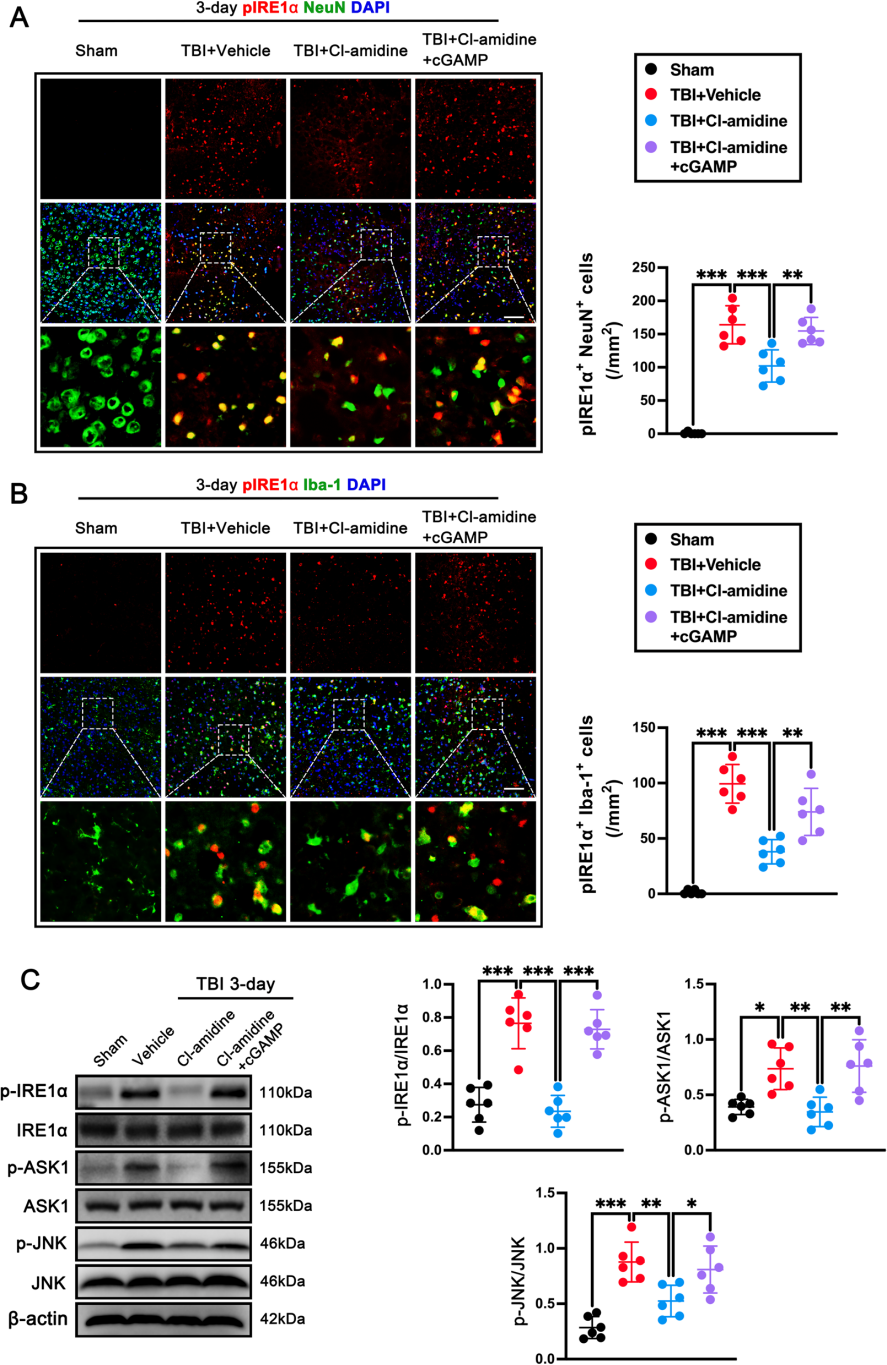
STING agonist abolished the neuroprotective effects of Cl-amidine via IRE1α/ASK1/JNK signaling pathway after TBI. **A** Representative double immunofluorescence staining for NeuN (green) and pIRE1α (red) and quantitative analyses of pIRE1α^+^ neuron in the contused cortex at 3 days after TBI. Nuclei were stained with DAPI (blue). Scale bar = 100 μm. **p < 0.01, ***p < 0.001, n = 6 per group. **B** Representative double immunofluorescence staining for Iba-1 (green) and pIRE1α (red) and quantitative analyses of pIRE1α^+^ microglia in the contused cortex at 3 days after TBI. Nuclei were stained with DAPI (blue). Scale bar = 100 μm. **p < 0.01, ***p < 0.001, n = 6 per group. **C** Representative western blot bands and densitometric quantification of pIRE1α, pASK1, and pJNK in the contused cortex after TBI. *p < 0.05, **p < 0.01, ***p < 0.001, n = 6 per group

To explore whether Cl-amidine exerts neuroprotective effects by regulating STING-dependent IRE1α/ASK1/JNK signaling pathway after TBI, immunofluorescence staining and western blot were performed. The results showed that Cl-amidine-treated TBI mice showed a significantly reduced number of IRE1α-positive neurons in mice treated with Cl-amidine when compared with TBI + Vehicle mice (p < 0.001, Fig. 8A), whereas 2′3′-cGAMP pretreatment reversed this effect of Cl-amidine (p < 0.01, Fig. 8A). Importantly, we also detected that the number of IRE1α-positive microglia significantly reduced in the contused cortex of mice treated with Cl-amidine when compared with that in TBI + Vehicle mice (p < 0.001, Fig. 8B). Similarly, 2′3′-cGAMP significantly increased the number of IRE1α-positive microglia when compared with TBI + Cl-amidine group at 3 days after TBI (p < 0.01, Fig. 8B). In line with these findings, western blot analyzes showed that the protein levels of p-IRE1α (p < 0.001, Fig. 8C), p-ASK1 (p < 0.05, Fig. 8C), and p-JNK (p < 0.001, Fig. 8C) were significantly increased in contused cortex when compared with the Sham group at 3 days after TBI. Cl-amidine post-treatment significantly decreased the expression of p-IRE1α (p < 0.001, Fig. 8C), p-ASK1 (p < 0.01 Fig. 8C), and p-JNK (p < 0.01, Fig. 8C) when compared with TBI + Vehicle group. However, pretreatment with 2′3′-cGAMP remarkably increased the expression of p-IRE1α (p < 0.001, Fig. 8C), p-ASK1 (p < 0.01 Fig. 8C), and p-JNK (p < 0.05, Fig. 8C) in contused cortex when compared with the TBI + Cl-amidine group at 3 days after TBI.

Taken together, our findings demonstrated that STING ligand 2′3′-cGAMP abolished neuroprotection of Cl-amidine via IRE1α/ASK1/JNK signaling pathway after TBI, indicating that NET may exacerbate neuroinflammation and neuronal death primarily via STING-dependent IRE1α/ASK1/JNK signaling pathway after TBI.

### PAD4 promotes neuroinflammation and neuronal death via IRE1α/ASK1/JNK signaling pathway at 3 days after TBI

**Fig. 9:**
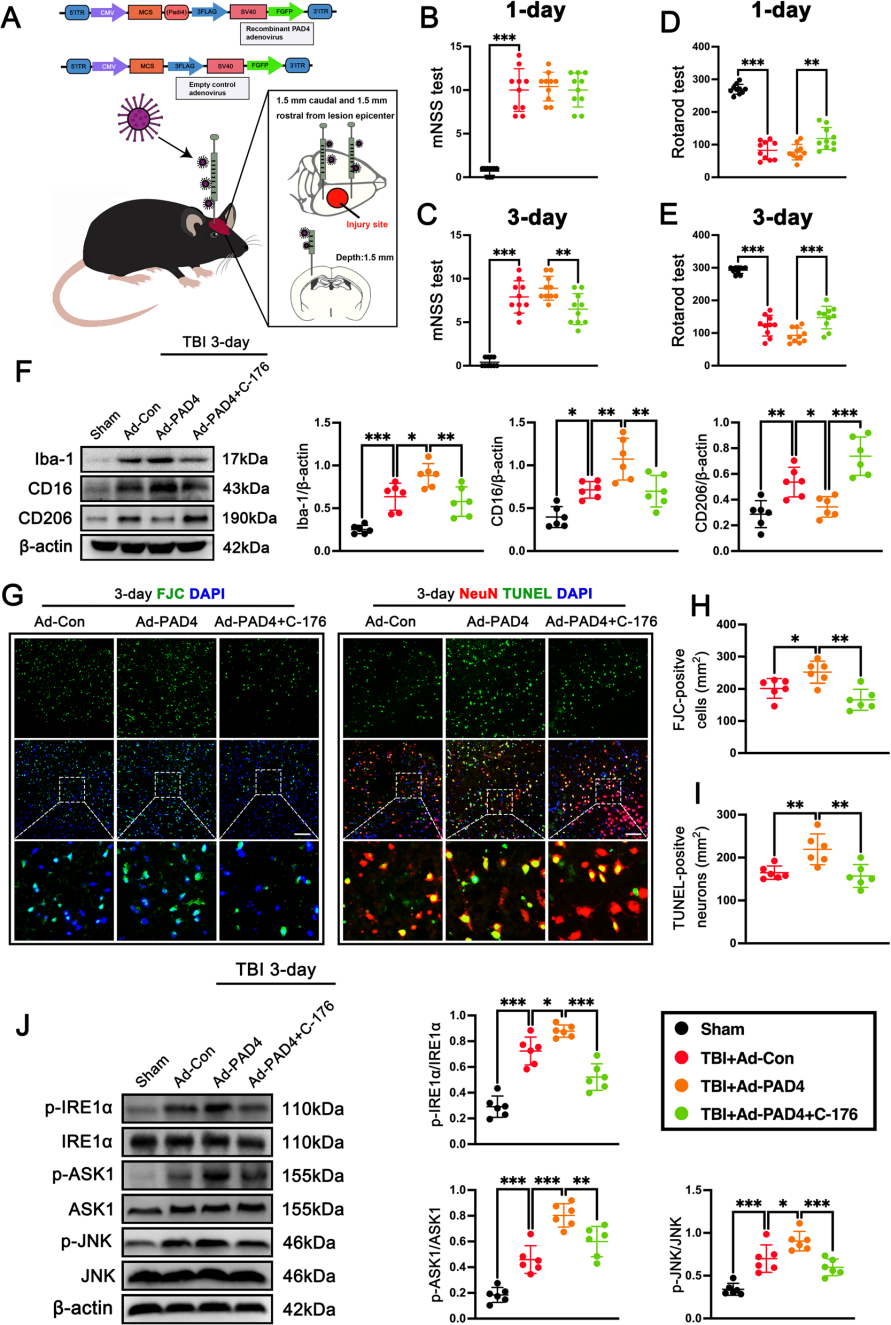
PAD4 promotes neuroinflammation and neuronal death via IRE1α/ASK1/JNK signaling pathway after TBI. **A** Schematic image of adeno-PAD4-EGFP and overexpression of PAD4 in the cortex of the mouse. **B**, **C** mNSS test on day 1 (**B**) and days 3 (**C**) post-TBI. **p < 0.01, ***p < 0.001, n = 10 per group. **D**, **E** Rotarod test on day 1 (**D**) and days 3 (**E**) post-TBI. *p < 0.05, **p < 0.01, ***p < 0.001, n = 10 per group.** F** Representative western blot bands and densitometric quantification of Iba-1, CD16, and CD206 in the contused cortex after TBI. *p < 0.05, **p < 0.01, ***p < 0.001, n = 6 per group. **G** Representative image of the FJC (green) staining and representative images of TUNEL (green) co-localization with neurons (NeuN, red) in the contused cortex at 3 days after TBI. Nuclei were stained with DAPI (blue). Scale bar = 100 μm. **H**, **I** Quantitative analysis of FJC-positive cells (**H**) and quantitative analysis of TUNEL-positive neurons (**I**) in the contused cortex at 3 days after TBI. *p < 0.05, **p < 0.01, n = 6 per group.** J** Representative western blot bands and densitometric quantification of pIRE1α, pASK1, and pJNK in the contused cortex after TBI. *p < 0.05, **p < 0.01, ***p < 0.001, n = 6 per group

To further test the hypothesis that increased formation of NET, orchestrated by PAD4, participated in microglia/macrophage-mediated inflammation and neuronal death after TBI, injections of adenovirus were completed to overexpress PAD4 in the vicinity of the cortex to be lesioned prior to the injury (Fig. 9A). In our time-course analysis of PAD4 overexpression, we observed a significant increase in PAD4 expression one day prior to traumatic brain injury (TBI) following viral administration (Additional file 1: Fig.S4A). Further western blot bands showed marked 2.28-fold upregulation of PAD4 protein expression (p < 0.01, Additional file 1: Fig. S4B) and 1.73-fold upregulation of H3Cit protein expression (p < 0.01, Additional file 1: Fig. S4C) in the contused cortex at 3 days after injection of PAD4 adenovirus (Ad-PAD4) when compare with control virus (Ad-Con) in mice with TBI. Consistently, we found that the H3cit-positive neutrophils in the cortex of Ad-PAD4-infected mice were significantly increased when compared with that in Ad-Con-infected mice at 3 days after TBI (p < 0.01, Additional file 1: Fig. S4D). These results indicate that NET formation in the contused cortex was increased after injection of PAD4 adenovirus.

Then, we found that pre-injection of PAD4 adenovirus cannot significantly aggravate the neurological function assessed by the mNSS test at 1 day (p = 0.958, Fig. 9B) and 3 days (p = 0.441, Fig. 9C), and Rotarod test at 1 day (p = 0.961, Fig. 9D) and 3 days (p = 0.074, Fig. 9E) in TBI + Ad-PAD4 mice when compared with TBI + Ad-Con mice. However, STING antagonist C-176 post-treatment significantly ameliorated neurological function assessed by the mNSS test at 3 days (p < 0.01, Fig. 9C), and Rotarod test at 1 day (p < 0.01, Fig. 9D) and 3 days (p < 0.001, Fig. 9E) when compared with TBI + PAD4 mice. Compared with the TBI + Ad Con group, we found that PAD4 overexpression significantly upregulated the expression of Iba-1 (p < 0.05, Fig. 9F) and M1 microglia marker CD16 (p < 0.01, Fig. 9F), while downregulated the proteins levels of M2 microglia marker CD206 (p < 0.05, Fig. 9F) in the contused cortex at 3 days after TBI. However, STING antagonist C-176 post-treatment significantly decreased the expression of Iba-1 (p < 0.01, Fig. 9F) and M1 marker CD16 (p < 0.01, Fig. 9F), while upregulated the proteins levels of M2 marker CD206 (p < 0.001, Fig. 9F**)** in the contused cortex at 3 days after TBI. Meanwhile, we also found that overexpression of PAD4 significantly increased the number of FJC-positive neurons (p < 0.05, Fig. 9G, H) and TUNEL-positive neurons (p < 0.01, Fig. 9G, I) in contused cortex when compared with TBI + Ad-Con group at 3 days after TBI. However, STING antagonist C-176 post-treatment significantly decreased the number of FJC-positive neurons (p < 0.01, Fig. 9G, H) and TUNEL-positive neurons (p < 0.01, Fig. 9G, I) in contused cortex when compared with TBI + Ad-PAD4 mice. Next, we explored whether STING-dependent IRE1α/ASK1/JNK signaling pathway was involved in PAD4-mediated neurodestructive effects after TBI. As the results showed, STING antagonist C-176 post-treatment significantly downregulated the protein levels of p-IRE1α (p < 0.001, Fig. 9J), p-ASK1 (p < 0.01 Fig. 9J), and p-JNK (p < 0.001, Fig. 9J) when compared with TBI + Ad-PAD4 group.

**Fig. 10:**
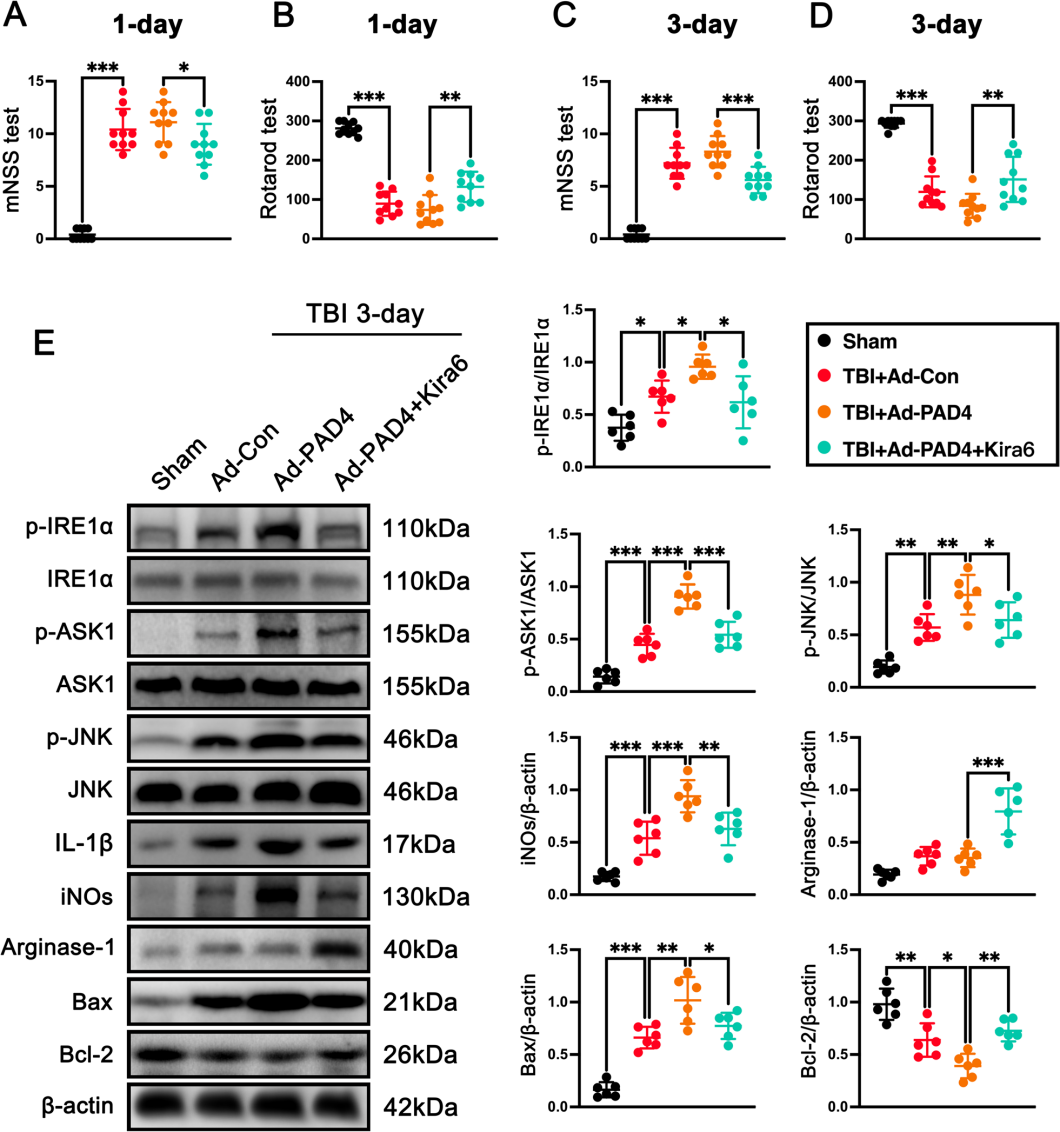
Inhibition of IRE1α abolished the neurodestructive effects caused by PAD4 overexpression after TBI. **A**, **B** mNSS test (**A**) and Rotarod test (**B**) at 1 day post-TBI. *p < 0.05, **p < 0.01, ***p < 0.001, n = 10 per group **C, D** mNSS test (**C**) and Rotarod test (**D**) at 3 days post-TBI. **p < 0.01, ***p < 0.001, n = 10 per group. **E** Representative western blot bands and densitometric quantification of pIRE1α, pASK1, pJNK, IL-1β, INOs, Arginase-1, Bax, and Bcl-2 after TBI. *p < 0.05, **p < 0.01, ***p < 0.001, n = 6 per group

To further confirm the key role of IRE1α in NETs-caused neuroinflammation and neuronal death after TBI, we used IREα inhibitor Kira6 after the injection of PAD4 adenovirus. First, we found that IREα inhibitor Kira6 post-treatment significantly ameliorated neurological function assessed by the mNSS test at 1 day (p < 0.05, Fig. 10A) and 3 days (p < 0.001, Fig. 10C), and Rotarod test at 1 day (p < 0.01, Fig. 10B) and 3 days (p < 0.01, Fig. 10D) when compared with TBI + Ad-PAD4 mice. The western blot analyses showed that Kira6 post-treatment significantly downregulated the protein levels of p-IRE1α (p < 0.05, Fig. 10E), p-ASK1 (p < 0.001, Fig. 10E), p-JNK (p < 0.05, Fig. 10E), iNOs (p < 0.01, Fig. 10E), Bax (p < 0.05, Fig. 10E), while upregulated the proteins levels of Arginase-1 (p < 0.001, Fig. 10E) and Bcl-2 (p < 0.01, Fig. 10E) in contused cortex when compared with TBI + Ad-PAD4 group.

Taken together, these results demonstrated that PAD4 exerted strong neurodestructive effects after TBI via STING-dependent IRE1α/ASK1/JNK signaling pathway at 3 days after TBI, further validating NET may exacerbate neurodestructive effects primarily via STING-dependent IRE1α/ASK1/JNK signaling pathway after TBI.

## Discussion

In the present study, we investigated the neuroprotective effects of NET formation inhibitor Cl-amidine-dependent STING activation and explored the underlying mechanism in a mouse model of TBI. For the first time, we demonstrated that activated neutrophils and neutrophil-derived NETs presented in the injured brain and peripheral blood after TBI. Furthermore, the inhibition of NET formation with Cl-amidine significantly improved the short-term neurobehavioral deficits, accompanied by alleviated BBB disruption, reduced brain edema, alleviated CBF reduction, attenuated microglia/macrophage activation, promoted microglia/macrophage phenotypic switch from pro-inflammatory to anti-inflammatory phenotypes, inhibited peripheral immune cells infiltration, and suppressed neuronal degeneration and apoptosis in the traumatic hemisphere after TBI. In addition, we also demonstrated that STING-dependent IRE1α/ASK1/JNK signaling pathway might participate in the neurodestructive effects of NETs and that Cl-amidine, a selective inhibitor of PAD4 (a key enzyme in chromatin decondensation during NET formation), could attenuate STING-dependent neuroinflammation and neuronal death after TBI. Importantly, we further found that overexpression of PAD4 with adenoviruses in the cortex aggravated neuroinflammation and neuronal death, and STING antagonist or IRE1α inhibitor effectively abolished the neurodestructive effects of PAD4 overexpression after TBI. Taken together, our findings suggest that inhibition of NET formation may alleviate neural damage after TBI, which was, at least in part, mediated by the promotion of the STING-dependent IRE1α/ASK1/JNK signaling pathway. The administration of Cl-amidine may serve as a promising therapeutic strategy against TBI-induced secondary injury after TBI.

Secondary brain injury is a complicated and interrelated pathological process after TBI, and neuroinflammatory response is one of the most important reactions involved [2–4]. During TBI onset, resident microglia/macrophages undergo activation and migration, and massive immune cells are infiltrated, eliciting a series of inflammatory cytokines release and leading to further BBB damage and neural damage [45]. Resident microglia/macrophages immediately respond to trauma through sensing (damage-associated molecular patterns, DAMPs), which then promote cytokine and chemokine release, thereby altering the CNS environment and providing a signal for neutrophil infiltration [46]. Although numerous research has suggested that neuroinflammation after TBI is deleterious, current clinical investigations all showed that anti-inflammatory therapy is limited and often ineffective for patients with TBI [45]. Indeed, there is even a CRASH trial that has proven that high-dose of inflammation suppression is detrimental to the prognosis of TBI patients [47]. Activated microglia/macrophages are polarized into two major phenotypes: the pro-inflammatory classically M1-like phenotype and the anti-inflammatory alternatively M2-like phenotype [48, 49]. Generally, classically activated microglia/macrophages (M1-like) release detrimental pro-inflammatory mediators, creating a vulnerable tissue microenvironment. In contrast, alternatively activated microglia/macrophage (M2-like) secrete protective anti-inflammatory mediators and trophic factors, facilitating tissue debris clearance and promoting remyelination [50]. In the current study, we demonstrated that inhibition of NET formation with Cl-amidine partially suppressed pro-inflammatory microglia/macrophage activation and promoted anti-inflammatory microglia/macrophage activation at 3 days after TBI, indicating that the inhibition of neuroinflammation by Cl-amidine is a promising therapeutic strategy for TBI treatment. It is now acknowledged that microglial response is highly dynamic and complex and a previous study showed that pro-inflammatory microglia/macrophage activation dominates 3 to 7 days after TBI, while anti-inflammatory microglia/macrophages had minute fluctuation during these days [33]. Thus, the microenvironment for effective tissue repair requires the temporal and spatial cooperation of both pro-inflammatory microglia/macrophages and anti-inflammatory microglia/macrophages, and the optimal timing switch of microglial/macrophage phenotypes should be found therapeutically.

NETs, which are lattices of extracellular double-stranded DNA combined with several components, including histones, neutrophil elastase, myeloperoxidase (MPO), and cathepsin, are released by neutrophils and form traps to kill pathogens [11]. However, growing evidence suggests that dysfunction of the inhibitory mechanism of NET and excess NET yield are also prominent pathogenic mechanisms that may lead to various diseases [11]. A recent study has reported on the presence of NETs in contused brain regions where their formation correlated with elevated intracranial pressure (ICP) and worse neurological function in patients with TBI [10]. Several experimental studies have reported that NETs have adverse effects on neurons, glia, and the blood–brain barrier, and then cause adverse consequences in central nervous system diseases [51, 52]. However, the contribution and underlying mechanisms of NETs to TBI-induced alteration of pathophysiology remain to be elucidated. Here, we demonstrated using co-localization of NET-specific markers (H3cit, MPO, MPO-DNA complexes, and DNA) that NETs are present in brain tissue and peripheral blood of mice with CCI. In the present study, we further detected that NETs inhibitor Cl-amidine effectively attenuated microglia-mediated neuroinflammation and neuronal death in mice subjected to TBI. This result is consistent with previous observations in mice with SAH where digestion of NETs with DNase 1 effectively inhibited the pro-inflammatory phenotype transition of microglia/macrophages and neuronal death [52]. DNases are part of the endogenous system to degrade NETs and they help to maintain tissue integrity during inflammation [53]. NETs as the main source of ds-DNA, we hypothesize that NET-derived DNA contributes to neuroinflammation, neuronal death, and blood–brain barrier disruption via the STING pathway [25, 37]. Notably, STING has been reported to be primarily expressed in neurons and microglia in the brain [54]. Consistently, our research found that STING was mainly expressed in neurons and microglia and STING-positive neurons and microglia were significantly increased in mice subjected to TBI. In addition, further investigation in our research revealed that inhibition of NET formation significantly attenuated neuroinflammation and neuronal death via STING in mice with TBI, whereas overexpression of peptidyl arginine deiminase 4 (a key enzyme for NET formation [55]) in the cortex by injection of adenoviruses could aggravate STING-dependent neuronal death and microglial activation. Collectively, these results indicated that NETs play a detrimental role in promoting neuronal death and microglia-mediated neuroinflammation in a STING-dependent manner after TBI.

Persistent ER stress and unfolded protein response (UPR) are associated with multiple pathophysiological processes of TBI including neuroinflammation, neuronal cell death, blood–brain barrier damage, axon injury, neurodegeneration, etc. [21]. IRE1α, as one of the key ER stress sensors, was reported to play an important role in neuronal death and microglial activation via TRAF2/ASK1/JNK signaling pathway after TBI [21, 56]. Here, we first found that inhibition of NET formation with Cl-amidine effectively reduced the expression of IRE1α in neurons and microglia following TBI and ameliorated neuronal apoptosis and microglia-mediated inflammatory responses by suppressing activation of STING-dependent IRE1α/ASK1/JNK signaling pathway. A recent study demonstrated that NET formation could accelerate intestinal epithelial cell death by activating ER stress activation-associated protein kinase RNA-like ER kinase (PERK) signaling pathways [57]. However, whether and how the NET formation is related to IRE1α-associated neural damage and neuroinflammation after TBI remains unclear. In the present study, we found that inhibition of NET formation significantly inhibited IRE1α activation-associated neuronal death and neuroinflammation in a STING-dependent manner after TBI. In our current research, STING, a transmembrane protein located in the ER, was demonstrated to act as a bridge between NET formation and IRE1α activation. Importantly, we found overexpression of PAD4 effectively aggravated IRE1α activation-associated neuronal apoptosis and neuroinflammation and IRE1α inhibitor Kira6 reversed the neurodestructive effects of PAD4 overexpression after TBI. These results demonstrated that NET formation is responsible for STING-mediated IRE1α activation in microglia and neurons, and inhibition of NET formation alleviated neural damage primarily via STING-dependent IRE1α/ASK1/JNK signaling pathway after TBI.

Several limitations of this study need to be discussed here. First, we did not evaluate the effects of Cl-amidine in different age groups or TBI with systemic comorbidities, and female animals. Further studies are needed to demonstrate the neuroprotective effects of Cl-amidine in experimental TBI in different age groups and females. Second, considering adenovirus vectors are inflammatory and immunogenic and can be cytotoxic at high multiplicities of infection in the nervous system [58], however, we did not test the inflammatory response of adenovirus infection in the informative controls, which would cause false positive effects on our results and confound the effects of PAD4 overexpression in the context of the acute inflammatory response of TBI. Further studies are needed to include a TBI group without adenovirus injection or a sham group with an adenovirus vector to exclude the potential effects and confounds of adenovirus injection in the context of the acute inflammatory response of TBI. Third, we only investigated the neuroprotective effect of Cl-amidine on neuroinflammation and neuronal death after TBI through the STING-dependent IRE1α/ASK1/JNK signaling pathway activation. Therefore, further studies are needed to explore the other mechanisms underlying the neuroprotective effects of NETs inhibition after TBI. In addition, our study demonstrates that STING inhibition can reduce FJC and TUNEL-positive cells, underscoring the significance of STING-associated pathology in contributing to neuronal death and neurodegeneration. However, it is crucial to recognize the multifaceted nature of TBI. Despite the positive effects of STING inhibition, it is evident that it does not fully restore these markers to uninjured levels, suggesting the involvement of other mechanisms in neuronal loss during the acute phase of TBI. As such, further investigations are warranted to elucidate these additional mechanisms that may underlie the neuroprotective effects of STING inhibition following TBI. Furthermore, in our present study, we employed two straightforward protein markers, CD16/32 and Arginase-1, for double-staining with Iba1 on microglia. Our observations revealed that Cl-amidine post-treatment facilitated a phenotypic shift of microglia/macrophages from the M1 pro-inflammatory phenotype to the M2 anti-inflammatory phenotype following TBI. Nonetheless, it’s essential to recognize that recent literature has cast doubt on the precision of this traditional classification. The microglial/macrophage response to brain injury is characterized by its remarkable dynamism and complexity. The oversimplified dichotomy between M1 and M2 phenotypes is increasingly viewed as an inadequate representation of the intricate and multifaceted nature of these immune cell responses. This emerging perspective highlights the need for a more nuanced understanding of microglial/macrophage phenotypes and behaviors in the context of brain injury. While our study provides valuable insights into this transition, it is essential to acknowledge that microglial/macrophage responses are highly dynamic and context-dependent. Future research in this field will continue to unveil the true complexity and relevance of these responses in neuroinflammation and TBI. Specifically, in future research, we will elucidate how our research findings contribute to the broader understanding of the complex nature of microglial responses and subpopulations in the contexts of neuroinflammation and traumatic brain injury (TBI). In line with this perspective and following the guidance provided in the “Neuron” expert consensus [59], we will refrain from employing the terms “M1” or “M2” and will instead use more precise terminology. Specifically, we will describe microglial responses in terms of their “inflammatory activity,” “regulatory activity,” and “immunomodulatory function,” and emphasize their “functional plasticity” and “dynamic immune responses.” Additionally, we will underscore the context-dependent nature of microglial phenotypes and their “functional heterogeneity.” Finally, we only investigate the role of NETs in neuronal death and microglial activation in vivo, additional in vitro experiments are also needed in the future to confirm the direct effects of NETs supplementation on STING-dependent neuronal death and microglial activation.

## Conclusion

In summary, we identified that NET inhibition with Cl-amidine alleviated neuroinflammation, neuronal apoptosis, and neurological deficits through STING-dependent IRE1α/ASK1/JNK signaling pathway in mice with TBI. Thus, inhibition of NET formation may be a potential therapeutic strategy for the management of TBI patients.

### Supplementary Information

**Additional file 1: Figure S1.** Schematic diagram of different experimental protocols and setups of this study. TBI, traumatic brain injury; NETs, neutrophil extracellular traps; WB, western blot; ELISA, enzyme-linked immunosorbent assay; qPCR, quantitative real-time polymerase chain reaction; IF, immunofluorescence; FJC, Fluoro-Jade C; TUNEL, terminal deoxynucleotidyl transferase dUTP nick-end labeling; BBB, blood-brain barrier; CBF, cerebral blood flow; mNSS, modified neurological severity score; MRI, magnetic resonance imaging; LSCI, laser speckle contrast imaging; Ad-PAD4, PAD4 adenovirus; Ad-con, empty adenovirus; PAD4, peptidyl arginine deiminase 4; IRE1α, inositol-requiring enzyme-1 alpha; ASK1, apoptosis signal-regulating kinase 1; JNK, c-Jun N-terminal kinase; i.p, intraperitoneal; i.v, intravenous. **Figure S2.** Time course of PAD4 after TBI and alteration of NETs after Cl-amidine treatment **Figure S3.** Time course and cellular localization of STING after TBI. **Figure S4.** Overexpression of PAD4 by adenovirus in the cortex increased NET formation in the cortex at 3 days after TBI. **Table S1.** Modified neurological severity scores. **Table S2.** Primers used for quantification of mRNA expression in the brain by RT-qPCR.

## Data Availability

The authors declare that all supporting data are available within the article and the Additional file data obtained under reasonable requirements.

## Abbreviations

TBI: Traumatic brain injury
ER: Endoplasmic reticulum
BBB: Blood–brain barrier
CBF: Cerebral blood flow
IL: Interleukin
NET: Neutrophil extracellular trap
DAMPs: Damage-associated molecular patterns
MPO: Myeloperoxidase
STING: Stimulating Interferon genes
IRE1α: Inositol-requiring enzyme-1 alpha
cGAS: Cyclic guanosine 5′-monophosphate (GMP)–adenosine 5′-monophosphate (AMP) synthase
dsDNA: Double-stranded DNA
GTP: Guanosine triphosphate
ATP: Adenosine triphosphate
2′3′-cGAMP: Cyclic GMP-AMP
TBK1: Tank-binding kinase 1
IRF3: Interferon regulatory factor 3
IFNs: Type I interferons
UPR: Unfolded protein response
TRAF2: TNF receptor-associated factor 2
ASK1: Apoptosis signal-regulating kinase 1
JNK: C-Jun N-terminal kinase
Ly6G: Anti-lymphocyte antigen 6 complex locus G
MPO: Myeloperoxidase
PAD4: Peptidylarginine deiminase 4
CCI: Controlled cortical impact
CNS: Central nervous system
DMSO: Dimethyl sulfoxide
Ad-PAD4: PAD4 adenovirus
Ad-Con: Empty adenovirus
mNSS: Modified neurological severity score
MRI: Magnetic resonance imaging
ELISA: Enzyme-linked immunosorbent assay
qPCR: 1Uantitative real-time polymerase chain reaction
SDS-PAGE: Sodium dodecyl sulfate–polyacrylamide gel electrophoresis
EB: Evans blue
OD: Optical density
LSCI: Laser speckle contrast imaging
PFA: Paraformaldehyde
FJC: Fluoro-Jade C
TUNEL: Terminal deoxynucleotidyl transferase dUTP nick-end labeling
i.p: Intraperitoneal
i.v: Intravenous
PBS: Phosphate-buffered saline
