# Supplementary material for: Inhibition of neutrophil extracellular trap formation ameliorates neuroinflammation and neuronal apoptosis via STING-dependent IRE1α/ASK1/JNK signaling pathway in mice with traumatic brain injury
Source: J Neuroinflammation. 2023 Sep 30;20:222. doi: 10.1186/s12974-023-02903-w (PMC10543875; doi:10.1186/s12974-023-02903-w)
Supplement: Supplementary file 1 — Additional file 1: Figure S1. Schematic diagram of different experimental protocols and setups of this study. TBI, traumatic brain injury; NETs, neutrophil extracellular traps; WB, western blot; ELISA, enzyme-linked immunosorbent assay; qPCR, quantitative real-time polymerase chain reaction; IF, immunofluorescence; FJC, Fluoro-Jade C; TUNEL, terminal deoxynucleotidyl transferase dUTP nick-end labeling; BBB, blood-brain barrier; CBF, cerebral blood flow; mNSS, modified neurological severity score; MRI, magnetic resonance imaging; LSCI, laser speckle contrast imaging; Ad-PAD4, PAD4 adenovirus; Ad-con, empty adenovirus; PAD4, peptidyl arginine deiminase 4; IRE1α, inositol-requiring enzyme-1 alpha; ASK1, apoptosis signal-regulating kinase 1; JNK, c-Jun N-terminal kinase; i.p, intraperitoneal; i.v, intravenous. Figure S2. Time course of PAD4 after TBI and alteration of NETs after Cl-amidine treatment Figure S3. Time course and cellular localization of STING after TBI. Figure S4. Overexpression of PAD4 by adenovirus in the cortex increased NET formation in the cortex at 3 days after TBI. Table S1. Modified neurological severity scores. Table S2. Primers used for quantification of mRNA expression in the brain by RT-qPCR. [file 12974_2023_2903_MOESM1_ESM.docx]

**Additional file**

**
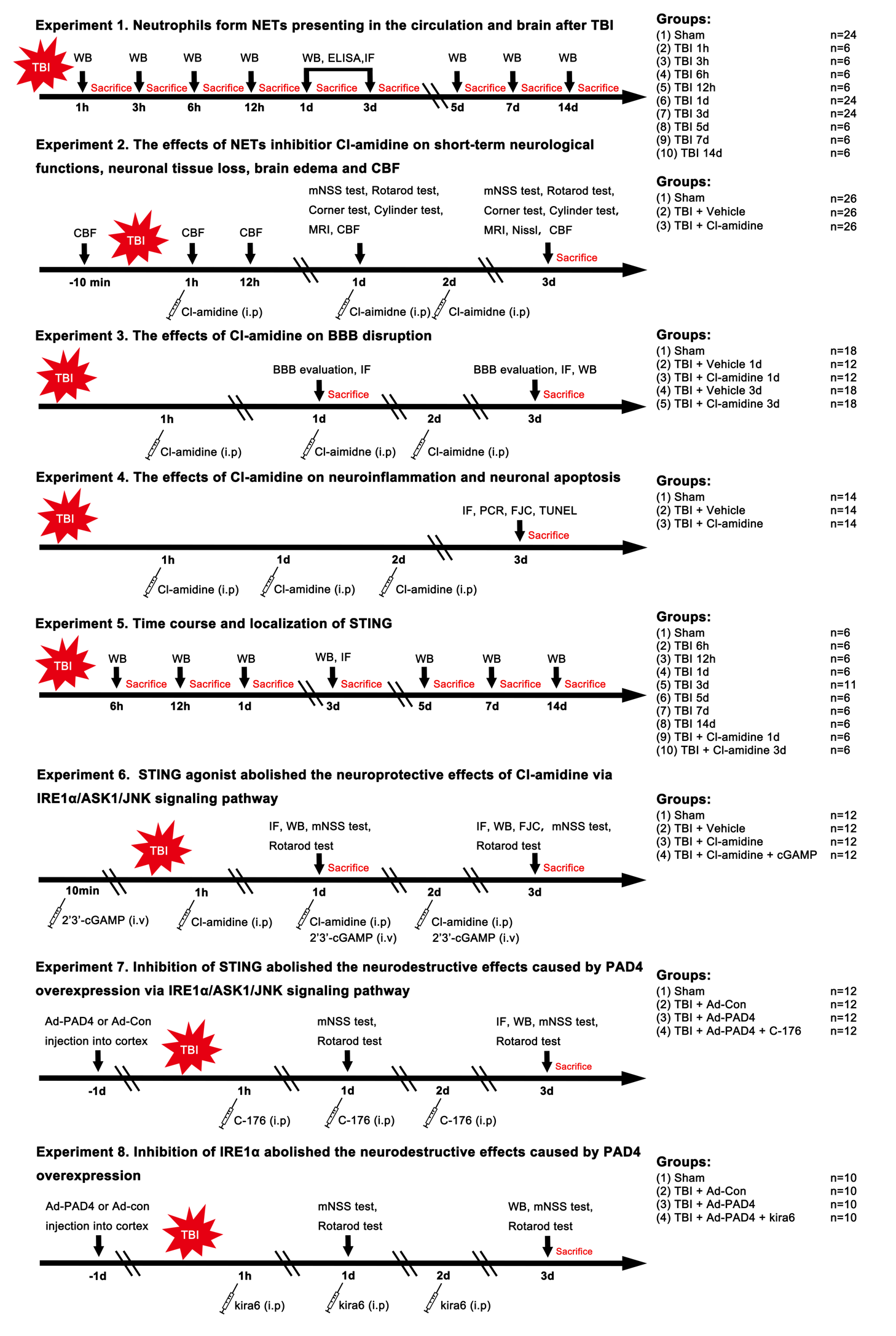
Part 1: Experimental design**

All mice were randomly assigned to the following experiments in the present study.

Figure S1. Schematic diagram of different experimental protocols and setups of this study. TBI, traumatic brain injury; NETs, neutrophil extracellular traps; WB, western blot; ELISA, enzyme-linked immunosorbent assay; qPCR, quantitative real-time polymerase chain reaction; IF, immunofluorescence; FJC, Fluoro-Jade C; TUNEL, terminal deoxynucleotidyl transferase dUTP nick-end labeling; BBB, blood-brain barrier; CBF, cerebral blood flow; mNSS, modified neurological severity score; MRI, magnetic resonance imaging; LSCI, laser speckle contrast imaging; Ad-PAD4, PAD4 adenovirus; Ad-con, empty adenovirus; PAD4, peptidyl arginine deiminase 4; IRE1α, inositol-requiring enzyme-1 alpha; ASK1, apoptosis signal-regulating kinase 1; JNK, c-Jun N-terminal kinase; i.p, intraperitoneal; i.v, intravenous.

**Experiment 1**

To evaluate the time course of Ly6G and H3cit expression in the contused cortex post-TBI, 60 mice were randomly assigned to ten groups (n = 6/group): 0 h (Sham), 1 h, 3 h, 6 h, 12 h, 1 d, 3 d, 5 d, 7 d, and 14 d after CCI for western blot analysis. To validate neutrophils-derived NETs were presented in the circulation and contused cortex post-TBI, an Enzyme-linked Immunosorbent Assay (ELISA) was performed to evaluate the plasma DNA concentration, plasma H3cit-DNA complexes, cortex H3cit-DNA complexes, and cortex MPO activity at 1 d and 3 d after TBI (n = 12/group). An additional six mice were used for double-labeling immunofluorescence staining to co-localize NETs (H3cit) with neutrophils (MPO) at 1 d and 3 d post-TBI.

**Experiment 2**

To determine the effects of peptidyl arginine deiminase 4 (PAD4) inhibitor Cl-amidine treatment on short-term neurological functions, brain edema, neuronal tissue loss, and cerebral blood flow (CBF) at 1d and 3d after TBI. First, to determine the optional dose of Cl-amidine after TBI in mice at 1d and 3d after TBI, 78 mice were randomly assigned into three groups: Sham, TBI + Vehicle, and TBI + Cl-amidine (n = 26/group). To test Cl-amidine post-treatment on mNSS test, Rotarod test, Corner test, and Cylinder test. 30 mice were randomly assigned into three groups: Sham, TBI + Vehicle, and TBI + Cl-amidine (n = 10/group). In addition, 24 mice were used for MRI and neuronal tissue loss after TBI (n = 8/group). In another set of experiments, additional 24 mice were randomly assigned into three groups (n = 8/group): Sham, TBI + Vehicle, and TBI + Cl-amidine and used for laser speckle contrast imaging (LSCI) after TBI.

**Experiment 3**

To investigate the effects of Cl-amidine on blood-brain barrier (BBB) disruption after TBI, a total of 78 mice were randomized into five groups: Sham (n=18/group), TBI 1 d + Vehicle (n=12/group), TBI 1d + Cl-amidine (n=12/group), TBI 3 d + Vehicle (n=18/group), TBI 3 d + Cl-amidine (n=18/group). Among these mice, 30 mice were randomly divided into five groups (n=6/group): Sham, TBI 1 d + Vehicle, TBI 1d + Cl-amidine, TBI 3 d + Vehicle, TBI 3 d + Cl-amidine for Evans blue assessment. 30 mice were randomly divided into five groups (n=6/group): Sham, TBI 1 d + Vehicle, TBI 1d + Cl-amidine, TBI 3 d + Vehicle, TBI 3 d + Cl-amidine for double immunofluorescence staining. The rest 18 mice were randomly divided into three groups (n=6/group): Sham, TBI 3 d + Vehicle, and TBI 3 d + Cl-amidine for western blot.

**Experiment 4**

To evaluate the effects of Cl-amidine on neuroinflammation and neuronal apoptosis at 3 d after TBI, a total of 42 mice were randomized into three groups (n=14/group): Sham, TBI 3 d + Vehicle, and TBI 3 d + Cl-amidine. Among these mice, 18 mice were randomly divided into three groups (n=6/group): Sham, TBI 3 d + Vehicle, and TBI 3 d + Cl-amidine for immunofluorescence staining and FJC staining. To further assess the effects of Cl-amidine on microglia/macrophage polarization, 24 mice were randomized into three groups (n=8/group) to perform quantitative real-time PCR (q-PCR): Sham, TBI 3 d + Vehicle, and TBI 3 d + Cl-amidine.

**Experiment 5**

To evaluate the time course and localization of STING expression in the contused cortex post-TBI, 48 mice were randomly assigned to seven groups (n = 6/group): 0 h (Sham), 6 h, 12 h, 1 d, 3 d, 5 d, 7 d, and 14 d after CCI for western blot analysis. Additional 5 mice were used for immunofluorescence and immunohistochemistry staining at 3 d post-TBI. The cellular localization of STING was assessed using double-labeling immunofluorescence staining to co-localize STING with neurons (NeuN), microglia (Iba-1), astrocytes (GFAP), neutrophils (Ly6G), and endothelial cells (CD31) at 3 d after TBI. To test the effects of Cl-amidine administration on the expression of STING in the contused cortex at 1 d and 3 d after TBI. 30 mice were randomly assigned to FIVE groups (n = 6/group): Sham, TBI 1 d + Vehicle, TBI 1d + Cl-amidine, TBI 3 d + Vehicle, TBI 3 d + Cl-amidine.

**Experiment 6**

To test the effects of Cl-amidine administration on neuroinflammation and neuronal death and to elucidate the underlying mechanism of neuroprotection of Cl-amidine after TBI, a total of 48 mice were randomized into four groups: Sham (n=12/group), TBI + Vehicle (n=12/group), TBI + Cl-amidine (n=12/group), and TBI + Cl-amidine +2′3′-cGAMP (n=12/group). 24 mice were randomly divided into four groups (n=6/group): Sham (n=6/group), TBI + Vehicle (n=6/group), TBI + Cl-amidine (n=6/group), and TBI + Cl-amidine +2′3′-cGAMP (n=6/group) for immunofluorescence staining and FJC staining. The rest 24 mice were randomly divided into four groups (n=6/group) for western blot at 3 d after TBI. Among these mice, 40 mice were subjected to the mNSS test and Rotarod test to evaluate short-term neurological function (n=10/group).

**Experiment 7**

To explore whether STING-dependent IRE1α/ASK1/JNK signaling pathway was involved in PAD4-mediated neurodestructive effects after TBI, a total of 48 mice were randomized into four groups: Sham (n=12/group), TBI + Ad-Con (n=12/group), TBI + Ad-PAD4 (n=12/group), and TBI + Ad-PAD4 + C-176 (n=12/group). Among these mice, half of them were used for immunofluorescence staining and a half for western blot. Among these mice, 40 mice were subjected to the mNSS test and Rotarod test to evaluate short-term neurological function (n=10/group).

**Experiment 8**

To further confirm the key role of IRE1α in NETs-caused neuroinflammation and neuronal death after TBI,

IREα inhibitor Kira6 was used. A total of 40 mice were randomized into four groups (n = 10/group): Sham (n = 10/group), TBI + Ad-Con (n=10/group), TBI + Ad-PAD4 (n=10/group), and TBI + Ad-PAD4 + C-176 (n=10/group) for Rotarod test and mNSS test, in which four groups including Sham (n = 6/group), TBI + Ad-Con (n=6/group), TBI + Ad-PAD4 (n=6/group), and TBI + Ad-PAD4 + C-176 (n=6/group) were used for western blot at 3 d after TBI.

**Part 2: Additional file tables**

Table S1. Modified neurological severity scores

| **Tests** | **Points** |
| --- | --- |
| **Motor tests (Normal score =0; maximum possible summary score = 6)**  **Raising the rat by the tail**  Flexion of forelimb  Flexion of hindlimb  Head moving >10° to vertical axis with 30 seconds  **Placing the rat on the floor**  Inability to walk straight  Circling toward the paretic side  Falling down to the paretic side | 1  1  1  1  1  1 |
| **Sensory tests (Normal score =0; maximum possible summary score = 2)**  Visual and tactile placing  Proprioceptive test (deep sensory) | 1  1 |
| **Beam balance tests** **(Normal score =0; maximum possible summary score = 6)**  Grasps the side of the beam  Hugs the beam and 1 limb falls down from the beam  Hugs the beam and 2 limbs fall down from the beam, or spins on the beam (< 60 seconds)  Attempts to balance on the beam but falls off (< 40 seconds)  Attempts to balance on the beam but falls off (< 20 seconds)  Falls off: no attempt to balance or hang on to the beam (< 20 seconds) | 1  2  3  4  5  6 |
| **Reflex absent and abnormal movement test (Normal score =0; maximum possible summary score = 4)**  Pinna reflex (a head shake when touching the auditory meatus)  Corneal reflex (an eye blink when lightly touching the cornea with cotton)  Startle reflex (a motor response to a brief loud paper noise)  Seizures, myoclonus, myodystony | 1  1  1  1 |
| Maximum points | 18 |

One point is awarded for the inability to perform the tasks or for the lack of a tested reflex; 13 to 18 indicates severe injury; 7 to 12 indicates moderate injury; 1 to 6 indicates mild injury[1].

| **Gene** | | **Sequences of primer (5’to 3’)** | |
| --- | --- | --- | --- |
| CD86 |  | Forward GGAACAACTGGACTCTACGAC  Reverse GTTTCGGGTGACCTTGCTTA | |
| iNOs |  | Forward GGAGCGAGTTGTGGATTGT  Reverse GTGGGAGGGGTCGTAATG | |
| TNF-α |  | Forward CCAGACCCTCACACTCAGATC  Reverse CACTTGGTGGTTTGCTACGAC | |
| IL-1β |  | Forward CCTGTGTTTTCCTCCTTGCCT  Reverse AGTGCGGGCTATGACCAATTC | |
| CD206 |  | Forward TGAGTCCAGTTTTCTGTCATACCGT  Reverse AGCCACTTCCCTTCAACATTTCG |  |
| Arginase-1 |  | Forward CTTGGCTTGCTTCGGAACTC  Reverse ACTGCTGGGATACATACTTACTGG |  |
| IL-10 |  | Forward ACTGCTATGCTGCCTGCTCTTAC  Reverse GCAACCCAAGTAACCCTTAAAGTCC |  |
| YM1/2 |  | Forward CCTGGACATGGATGACTTC  Reverse GGGATTCAATTTAGGAAAGTTCA |  |
| GAPDH |  | Forward AATGTGTCCGTCGTGGATCTGA  Reverse GATGCCTGCTTCACCACCTTCT |  |

Table S2. Primers used for quantiﬁcation of mRNA expression in the brain by RT-qPCR

**Part 3:** **Additional file figures**

**
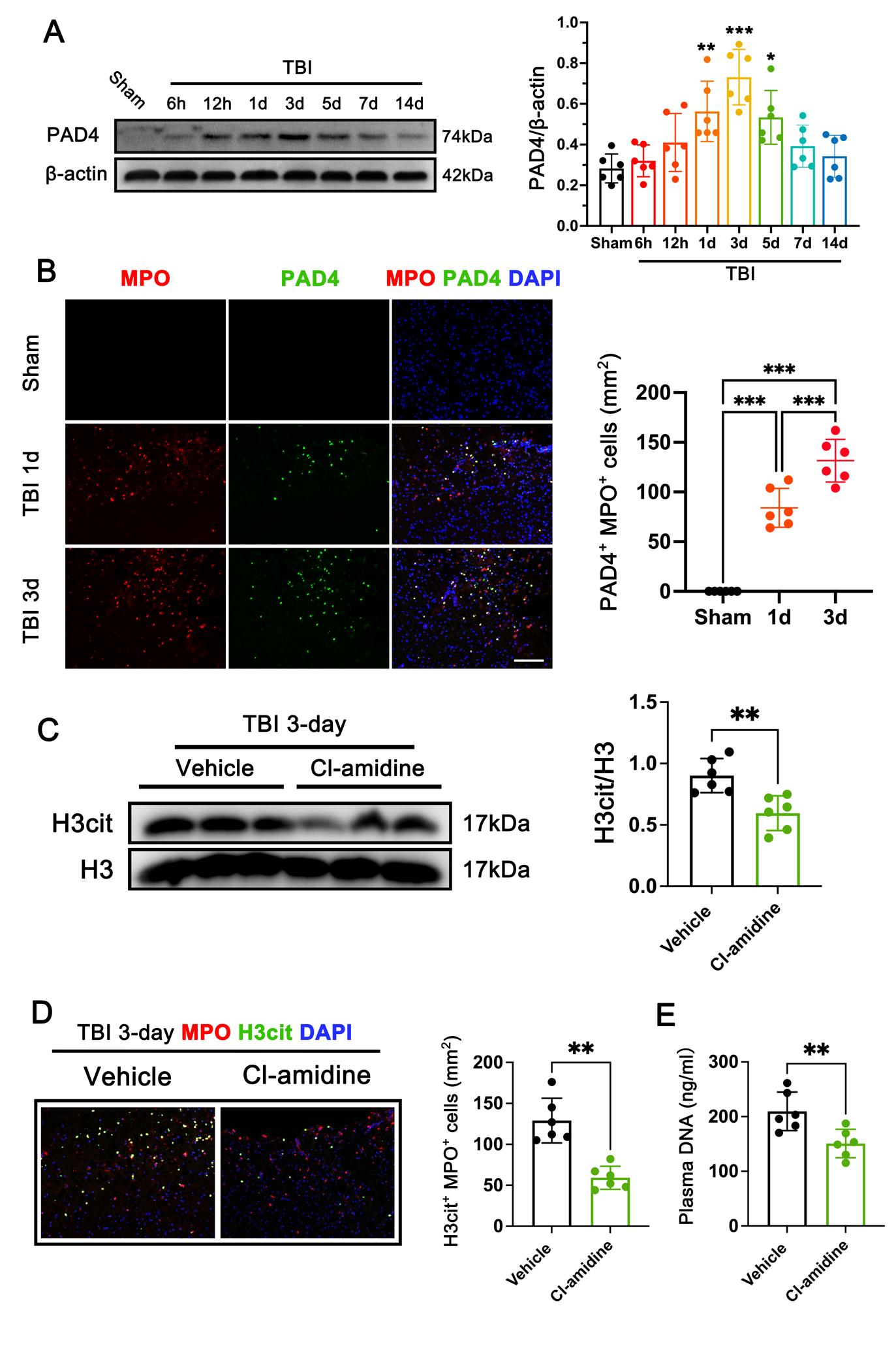
Figure S2. Time course of PAD4 after TBI and alteration of NETs after Cl-amidine treatment.**

Figure S2. **A** Representative western blot bands of the time course of PAD4 and densitometric quantification of PAD4 after TBI. *p < 0.05, **p < 0.01, ***p < 0.001, n = 6 per group. **B** Representative double immunofluorescence images of PAD4 (green) and neutrophils (MPO, red) and quantitative analysis of PAD4-positive neutrophils in the contused cortex at 1 d and 3 d after TBI. ***p < 0.001, n = 6 per group. Nuclei were stained with DAPI (blue). Scale bar = 100 μm. **C** Representative western blot bands and densitometric quantification of H3cit after TBI. **p < 0.01, n = 6 per group. **D** Representative double immunofluorescence images of H3cit (green) and neutrophils (MPO, red) and quantitative analysis of H3cit-positive neutrophils in the contused cortex at 3 d after TBI. **p < 0.01, n = 6 per group. Nuclei were stained with DAPI (blue). Scale bar = 100 μm**. E** Quantitative analyses of plasma DNA at 3 d after TBI. **p < 0.01, n = 6 per group

**Figure S3. Time course and cellular localization of STING after TBI.
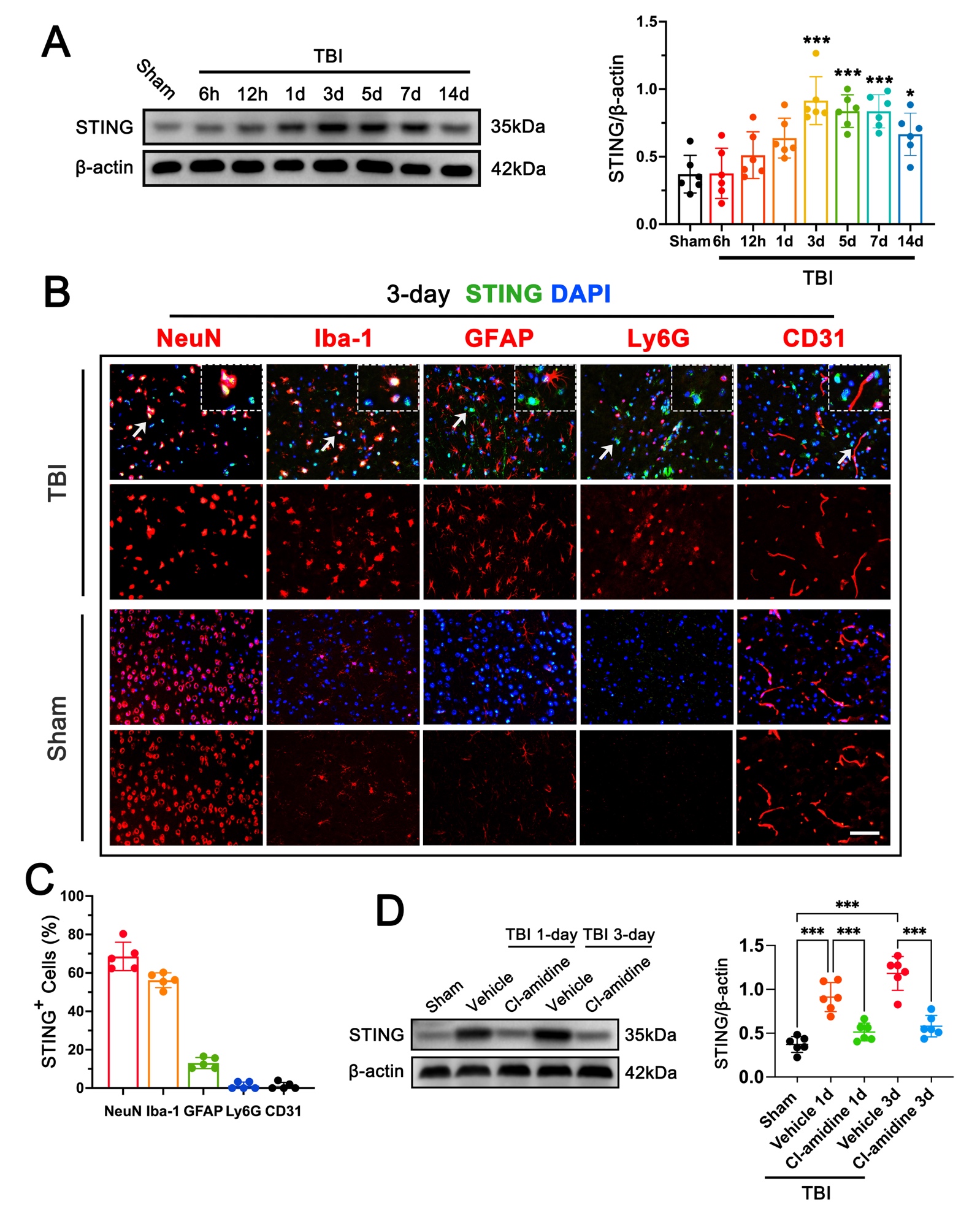
**

Figure S3. **A** Representative western blot bands of the time course of STING and densitometric quantification of STING after TBI. *p < 0.05, ***p < 0.001, n = 6 per group. **B** Representative images of the colocalization of STING (green) with neurons (NeuN, red), microglia (Iba1, red), astrocytes (GFAP, red), neutrophils (Ly6G, red), endotheliocytes (CD31, red) at the lesion site at 3 d after TBI. Nuclei were stained with DAPI (blue). Scale bar = 50 μm. **C** Quantitative analysis of the percentage of STING-positive neurons to neurons, STING-positive microglia to microglia, STING-positive astrocytes to astrocytes, STING-positive neutrophils to neutrophils, and STING-positive endotheliocytes to endotheliocytes. n = 5 per group. **D** Representative western blot bands and densitometric quantification of STING after TBI. ***p < 0.001, n = 6 per group.

**Figure S4: Overexpression of PAD4 by adenovirus in the cortex increased NET formation in the cortex at 3 d after TBI.**


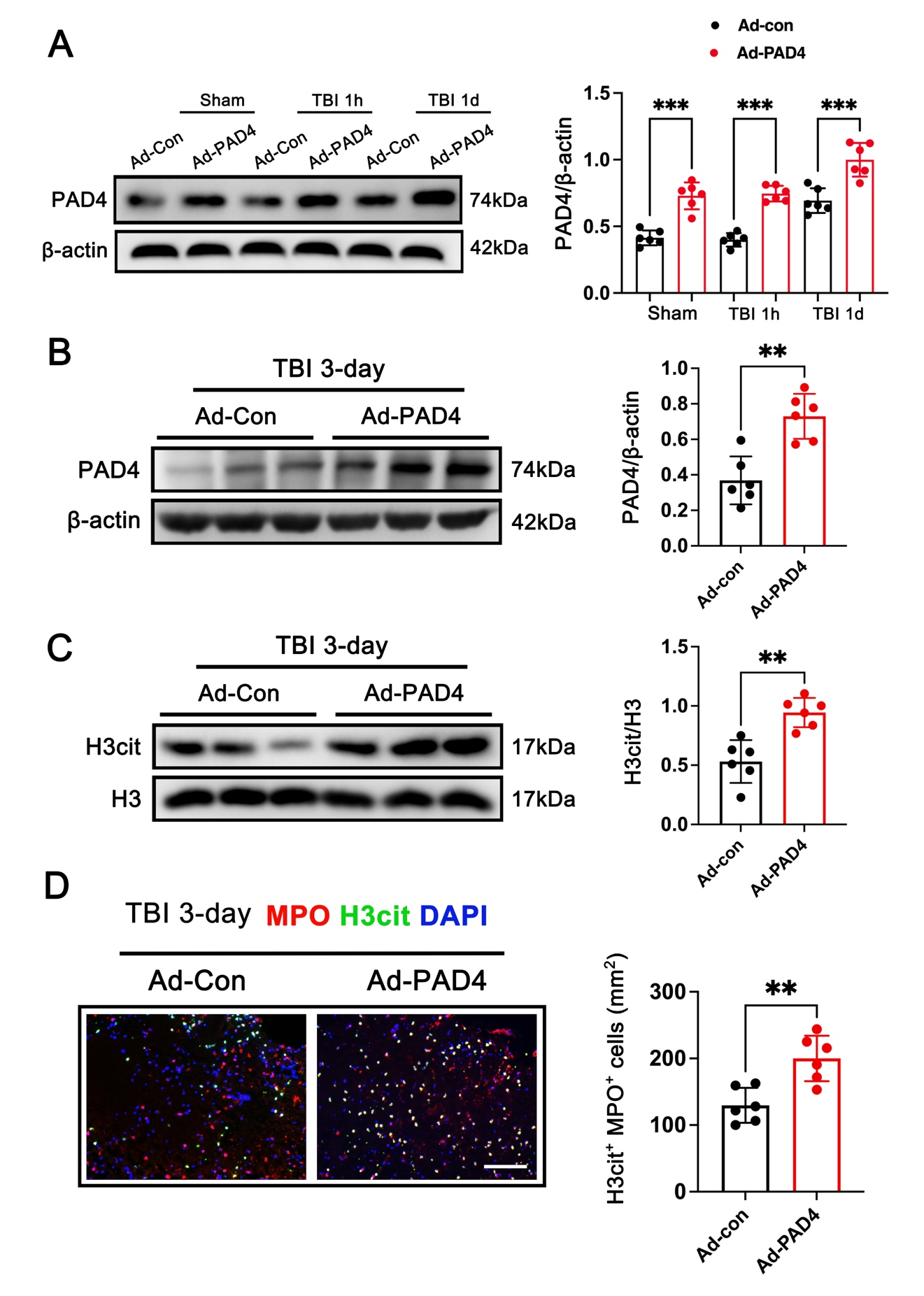


Figure S4. **A** Representative western blot bands and densitometric quantification of PAD4 after TBI. **p < 0.01, n = 6 per group. **B** Representative western blot bands and densitometric quantification of PAD4 after TBI. ***p < 0.001, n = 6 per group. **C** Representative western blot bands and densitometric quantification of H3cit after TBI. **p < 0.01, n = 6 per group. **D** Representative double immunofluorescence images of H3cit (green) and neutrophils (MPO, red) and quantitative analysis of H3cit -positive neutrophils in the contused cortex at 3 d after TBI. n = 6 per group. Nuclei were stained with DAPI (blue). Scale bar = 100 μm.

1. Chen, J., et al., *Therapeutic benefit of intravenous administration of bone marrow stromal cells after cerebral ischemia in rats.* Stroke, 2001. **32**(4): p. 1005-11.
